# Cholecystokinin-like peptide mediates satiety by inhibiting sugar attraction

**DOI:** 10.1371/journal.pgen.1009724

**Published:** 2021-08-16

**Authors:** Di Guo, Yi-Jie Zhang, Su Zhang, Jian Li, Chao Guo, Yu-Feng Pan, Ning Zhang, Chen-Xi Liu, Ya-Long Jia, Chen-Yu Li, Jun-Yu Ma, Dick R. Nässel, Cong-Fen Gao, Shun-Fan Wu

**Affiliations:** 1 College of Plant Protection, Nanjing Agricultural University, Nanjing, China/State & Local Joint Engineering Research Center of Green Pesticide Invention and Application, Jiangsu, China; 2 The Key Laboratory of Developmental Genes and Human Disease, Institute of Life Sciences, Southeast University, Nanjing, China; 3 School of Life Sciences, Tsinghua-Peking Joint Center for Life Sciences, IDG/McGovern Institute for Brain Research, Tsinghua University, Beijing, China; 4 Department of Zoology, Stockholm University, Stockholm, Sweden; Katholieke Universiteit Leuven, BELGIUM

## Abstract

Feeding is essential for animal survival and reproduction and is regulated by both internal states and external stimuli. However, little is known about how internal states influence the perception of external sensory cues that regulate feeding behavior. Here, we investigated the neuronal and molecular mechanisms behind nutritional state-mediated regulation of gustatory perception in control of feeding behavior in the brown planthopper and *Drosophila*. We found that feeding increases the expression of the cholecystokinin-like peptide, sulfakinin (SK), and the activity of a set of SK-expressing neurons. Starvation elevates the transcription of the sugar receptor Gr64f and SK negatively regulates the expression of Gr64f in both insects. Interestingly, we found that one of the two known SK receptors, CCKLR-17D3, is expressed by some of Gr64f-expressing neurons in the proboscis and proleg tarsi. Thus, we have identified SK as a neuropeptide signal in a neuronal circuitry that responds to food intake, and regulates feeding behavior by diminishing gustatory receptor gene expression and activity of sweet sensing GRNs. Our findings demonstrate one nutritional state-dependent pathway that modulates sweet perception and thereby feeding behavior, but our experiments cannot exclude further parallel pathways. Importantly, we show that the underlying mechanisms are conserved in the two distantly related insect species.

## Author summary

Food intake is critical for animal survival and reproduction and is regulated both by internal states that signal appetite or satiety, and by external sensory stimuli. It is well known that the internal nutritional state influences the strength of the chemosensory perception of food signals. Thus, both gustatory and olfactory signals of preferred food are strengthened in hungry animals. However, the molecular mechanisms behind satiety-mediated modulation of taste are still not known. We show here that cholecystokinin-like (SK) peptide in brown planthopper and *Drosophila* signals satiety and inhibits sugar attraction by lowering the activity of sweet-sensing gustatory neurons and transcription of a sugar receptor gene, *Gr64f*. We show that SK peptide signaling reflects the nutritional state and inhibits feeding behavior. Re-feeding after starvation increases SK peptide expression and spontaneous activity of SK producing neurons. Interestingly, we found that SK peptide negatively regulates the expression of the sweet gustatory receptor and that activation of SK producing neurons inhibits the activity of sweet-sensing gustatory neurons (GRNs). Furthermore, we found that one of the two known SK peptide receptors is expressed in some sweet-sensing GRNs in the proboscis and proleg tarsi. In summary, our findings provide a mechanism that is conserved in distantly related insects and which explains how feeding state modulates sweet perception to regulate feeding behavior. Thus, we have identified a neuropeptide signal and its neuronal circuitry that respond to satiety, and that regulate feeding behavior by inhibiting gustatory receptor gene expression and activity of sweet sensing GRNs.

## Introduction

Neuronal control of feeding is interesting for at least two reasons: in the human population there is a growing problem with excess food consumption causing obesity and associated severe health problems, and secondly, pest insects consume large amounts of our crops worldwide. Both problems are very costly to society. Therefore, understanding mechanisms behind regulation of food search, feeding and satiety is of great interest. In general, animal behavior is guided by internal states and external stimuli [1–5]. Hence, behavioral decisions depend on the integration of signals from the internal and external environment by circuits of the brain. For instance, feeding behavior, which is essential for the survival and reproduction of animals, is regulated by the nutritional state of the organism and depends on the efficacy of chemosensory organs in localizing food in the environment [3,6–12]. Thus, internal nutrient sensors monitor the energy homeostasis and signal hunger or satiety to the nervous system. Hunger signals received by brain circuits are relayed to sense organs to increase their sensitivity. Hence, in a hungry animal the sensory threshold is lowered in olfactory and gustatory receptors that respond to food cues and thereby increases appetitive behavior and food seeking [6,10–13]. Concomitantly, the hunger signals increase the detection threshold for aversive stimuli, such as bitter tastants [11,14]. After food ingestion, satiety signals lower the attractive sensory thresholds and also act on neuronal circuits that regulate feeding behavior, thereby stopping further food intake [12,15].

In *Drosophila* and other insects, the modulation of sensory gain in appetitive behavior is largely dependent on neuropeptides and peptide hormones [4,12,15–17]. These signaling systems also orchestrate animal behavior and link internal and external sensory cues [see [17–19]]. Thus, several neuropeptides are known to trigger appetitive behavior, foraging, and mobilize energy stores, at the same time as they suppress other conflicting behaviors such as sleep and reproductive behavior [see [17–20]]. At the sensory level neuropeptides such as short neuropeptide F (sNPF), myoinhibitory peptide (MIP), CCH2amide and tachykinin (TK) regulate the sensitivity of subpopulations of olfactory receptor neurons (ORNs) to promote food seeking in hungry flies [6,10,21–24]. Also gustatory neurons in *Drosophila* are modulated by neuropeptides to regulate sugar and bitter sensitivities [11]. Hence, in hungry flies neuropeptide F (NPF), via dopamine cells, increases sweet sensitivity in Gr5a expressing cells and sNPF decreases bitter sensitivity [11]. NPF and sNPF are also known to regulate aspects of feeding, metabolism and sleep [25–32], suggesting action at several levels of the organism. Another neuropeptide known to regulate food intake in *Drosophila* is drosulfakinin, DSK, related to the mammalian peptide cholecystokinin (CCK) [33]. CCK in mammals and sulfakinins (SKs) in insects, including *Drosophila*, signal satiety and decrease food ingestion [34–38]. DSK has furthermore been reported to modulate aggression and courtship behavior in *Drosophila* [39–41]. The first insect SK was identified from head extracts of the cockroach, *Rhyparobia maderae* [42]. Characteristic of SKs is that it contains a sulfated tyrosine residue (DY(SO3)GHM/LRFamide) [43]. In a variety of insects, such as the desert locust, the German cockroach, and the red flour beetle, SK significantly reduced food intake [44–46]. SK is also known to inhibit the activity of digestive enzymes in the migratory locust and brown planthopper [35,47].

In *Drosophila*, upon feeding, DSK is released and the meal is terminated [12,15,33]. However, it is not known how DSK acts to decrease feeding. The mechanisms controlling sweet gustation and feeding in insects are remarkably similar to those in vertebrates. Sweet-sensing neurons are housed in taste sensilla in the labellum on the proboscis (the insect equivalent of the vertebrate tongue), the tarsal segments of the legs, and the pharynx [48–50]. In *Drosophila*, nine types of gustatory receptors (Gr) participate in sweet sensation. One of them, Gr64f, is a co-receptor that is required in combination with other gustatory receptors for sugar detection in *Drosophila* [49–51]. However, the mechanisms behind how the feeding state modulates sugar receptor gene expression and sweet-sensing neurons activity are poorly known.

We chose to study SK signaling in regulation of gustatory input and feeding behavior in two insect species, the brown planthopper *Nilaparvata lugens* and the genetic model insect *Drosophila melanogaster*. The brown planthopper is a serious pest on rice in Asia and causes damage costing more than 300 million US dollars annually [52]. *N*. *lugens* is a monophagous pest that pierce the rice stem and sucks sap, thereby transferring a virus [53] that destroys the plant [54]. With the magnificent genetic toolbox available, *Drosophila* has been extensively used as a model to study regulation of feeding, metabolism and sensory mechanisms underlying food seeking [see [4,12,13,15,17,55]]. Thus, we combine two insect species in our quest to understand how a neuropeptide regulates gustatory perception of food-related taste and ensuing initiation of food ingestion.

Using *N*. *lugens* in an RNA-seq transcriptome screen for altered gene expression after downregulation of SK, we found among others an upregulation of sweet-sensing gustatory receptors and the *takeout (to)* gene. Analysis of manipulations of SK and its receptor SKR, as well as gustatory receptors and *to*, suggests that feeding-induced SK signaling downregulates sweet sensing receptors and that *to* is a mediator of SK signaling in the planthopper. Further experiments in *Drosophila* unravel mechanisms behind the satiety signaling that modulates gustatory neurons. Feeding upregulates *Dsk* transcription and increases spontaneous activity and calcium signaling in DSK expressing median protocerebrum (MP) neurons. Furthermore, feeding downregulates Gr64f expression and starvation increases Gr64f. Optogenetic activation of Gr64f neurons increases the motivation to feed. In addition to this, knockdown of *dsk* leads to an upregulation of Gr64f transcription and activation DSK expressing MP neurons inhibit the sensitivity of gustatory neurons. Remarkably, we found expression of the DSK receptor CCKLR-17D3 in a subpopulation of the Gr64f GRNs in proleg tarsi, proboscis and maxillary palps and this receptor is downregulated in the appendages after feeding. Finally, knockdown of 17D3 in sweet sensing GRNs decreases the PER. Thus, in summary DSK signaling modulates the sensitivity of sweet-sensing GRNs in a nutrient-dependent fashion in both the planthopper and *Drosophila*, suggesting a conserved peptidergic signal pathway in these distantly related insects. It should be emphasized that our experiments cannot exclude further components in the state-dependent regulation of gustatory sensitivity, including contributions of other neuropeptides or neuromodulators, such as those indicated earlier in this introduction.

## Results

### Sulfakinin (SK) reduces food intake in *N*. *lugens*

**Fig 1:**
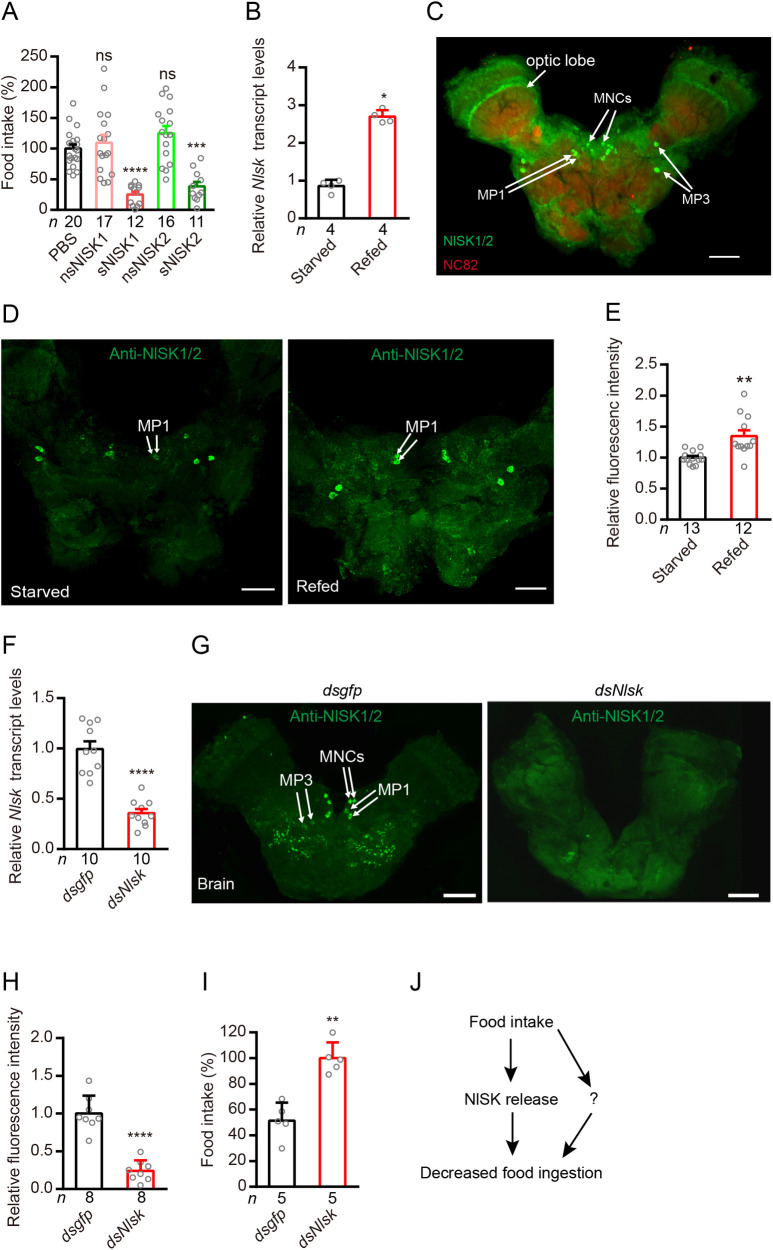
Sulfakinin (NlSK) and its receptor (SKR) signal satiety and inhibit feeding in the rice planthopper. (**A**) Injection of *N*. *lugens* sulfated sulfakinins inhibits food intake in the brown planthopper. Fifty nanoliter of PBS (as control) and four sulfakinins (20 pmol/insect) were injected into 4^rd^ instar nymph of brown planthopper. Non-sulfated Sulfakinins (nsNlSK1 and 2) have no effect. All data are presented as means ± s.e.m in this manuscript. ns: not significant, ***p < 0.001, ****p < 0.0001; One-way ANOVA followed by Tukey’s multiple comparisons test. (**B**) Refeeding for 5 hr after 24 hr starvation increases *Nlsk* mRNA transcript. *p < 0.05; Mann–Whitney test. **(C)** NlSK expression in the brain is revealed by anti-NlSK1/2 (green) and anti-nc82 (red). Two pairs of MP1 (medial protocerebrum), two pairs of MP3 neurons, and median neurosecretory cells (MNCs) are indicated. Bar: 50 μm. (**D and E**) Refeeding after 24h starvation increases NlSK peptide expression as revealed by anti-NlSK1/2 immunolabeling. Scale bars, 50 μm. **p < 0.01; Student’s *t* test. Note that we measure SK levels in neuronal MP1 cell bodies where production occurs. (**F**) Downregulation of *Nlsk* gene using *Nlsk*-RNAi leads to a reduction in mRNA expression level. ****p < 0.0001; Student’s *t* test. (**G and H**) Downregulation of *Nlsk* gene using *Nlsk*-RNAi (*dsNlsk*) leads to a reduction in NlSK1/2 immunoreactivity. In (G) we show NlSK1/2 immunoreactivity in brains of *dsgfp*- or *dsNlsk*-injected planthoppers. Scale bar: 50 μm. (H) Intensity of anti-NlSK1/2 immunoreactivity in brain. All data are presented as means ± s.e.m. ****p < 0.0001; Student’s *t* test. (**I**) Downregulation of *Nlsk* gene using *Nlsk*-RNAi (*dsNlsk*) increases the food intake. **p < 0.01; Mann–Whitney test. (**J**) Simplified model showing that NlSK inhibits feeding. Note that this model and those in subsequent figures of planthopper data are highly simplified and serve to summarize our data, rather that presenting accurate signaling pathways. The question mark indicates one or more possibly additional signals.

In several insect species, SK is known to inhibit feeding and act as a satiety factor [33–35,44–46,56,57]. To test whether SK induces satiety also in *N*. *lugens*, we injected 4^th^ instar nymph with 20 pmol of either of four types of *N*. *lugens* sulfakinins (NlSKs), namely nsNlSK1 (non-sulfated SK1), sNlSK1 (sulfated SK1), nsNlSK2 (non-sulfated SK2) and sNlSK2 (sulfated SK2), dissolved in PBS. After 24h we compared the food uptake to a control group, which was injected with PBS. *N*. *lugens* injected with sNlSK1 or sNlSK2 consumed 50%-70% less food than the PBS-injected control. However, non-sulfated SKs (nsNlSK1 and nsNlSK2) had no impact on the feeding behavior of *N*. *lugens* (Fig 1A).

### Effects of *Nlsk* gene silencing on food intake

Next, we asked whether the gene expression level of *Nlsk* is affected by the satiety state. Indeed, we found that the *Nlsk* mRNA level is significantly higher in brown planthoppers that had been refed after 24 h starvation, compared to starved ones (Fig 1B). We furthermore examined the expression pattern of the NlSK peptides in the brain using anti-NlSK1/2 (Fig 1C). NlSK1/2 immunoreactivity was observed in the median neurosecretory cells (MNCs) as previously reported in *Drosophila* [33], as well as in two pairs of cells in the median protocerebrum (MP1 and MP3) also shown earlier in *Drosophila* [41,58]. We then asked whether refeeding leads to more NISK release from neurons. Our data showed that refeeding increases the immunolabeling intensity in the cell bodies of NlSK MP1 neurons compared with starved animals (Fig 1D and 1E). This might be explained by increased SK production accompanying the release at axon terminations. To determine the role of the *Nlsk* gene in feeding behavior, we performed RNAi injection experiments. The efficacy of the RNAi was tested by qPCR of whole animals and we found that the expression of *Nlsk* was significantly reduced (Fig 1F). The NlSK1/2 immunoreactivity in the brain was also significantly reduced by the RNAi (Fig 1G and 1H). Feeding experiments showed that the *dsNlsk*-injected planthoppers consumed approximately 2-fold more food than the *dsgfp*-injected controls (Fig 1I). Taken together, these results show that NlSK signaling reflects the nutritional state of the animal and inhibits feeding behavior (Fig 1J). However, we cannot rule out there are other neuromodulators that also play important roles in the satiety-induced regulation of food ingestion.

### Global gene expression profiling of *N*. *lugens* in response to *Nlsk* gene-silencing

To unravel the molecular mechanisms underlying control of feeding behavior by *Nlsk* signaling, we performed transcriptome expression profiling (RNA-seq quantification) of *N*. *lugens* after *Nlsk* knockdown by *dsNlsk* injection. Illumina sequencing libraries were constructed by using mRNA from the *dsNlsk*- or *dsgfp* (control)-injected 4^th^ instar brown planthoppers. We obtained 49,055,819.5 and 47,254,495 clean reads on average from samples of *Nlsk*–depleted and control groups, respectively (S2 Table). After removing low-quality regions, adapters, and possible contamination, we obtained more than 6 giga base clean bases with a Q20 percentage over 96%, Q30 percentage over 92%, and a GC percentage between 49.12 and 51.53% (S2 Table). After alignment by Bowtie, 61.01–65.66% and 61.97–66.21% unique reads were mapped into the reference genome of *N*. *lugens* (S3 Table). For non-model insects, this level of unique reads coverage (over 60%) is acceptable for further analysis. All of the RNA sequence data in this article have been deposited in the NCBI SRA database and are accessible in PRJNA657327 (SRR12460889-96).

**Fig 2:**
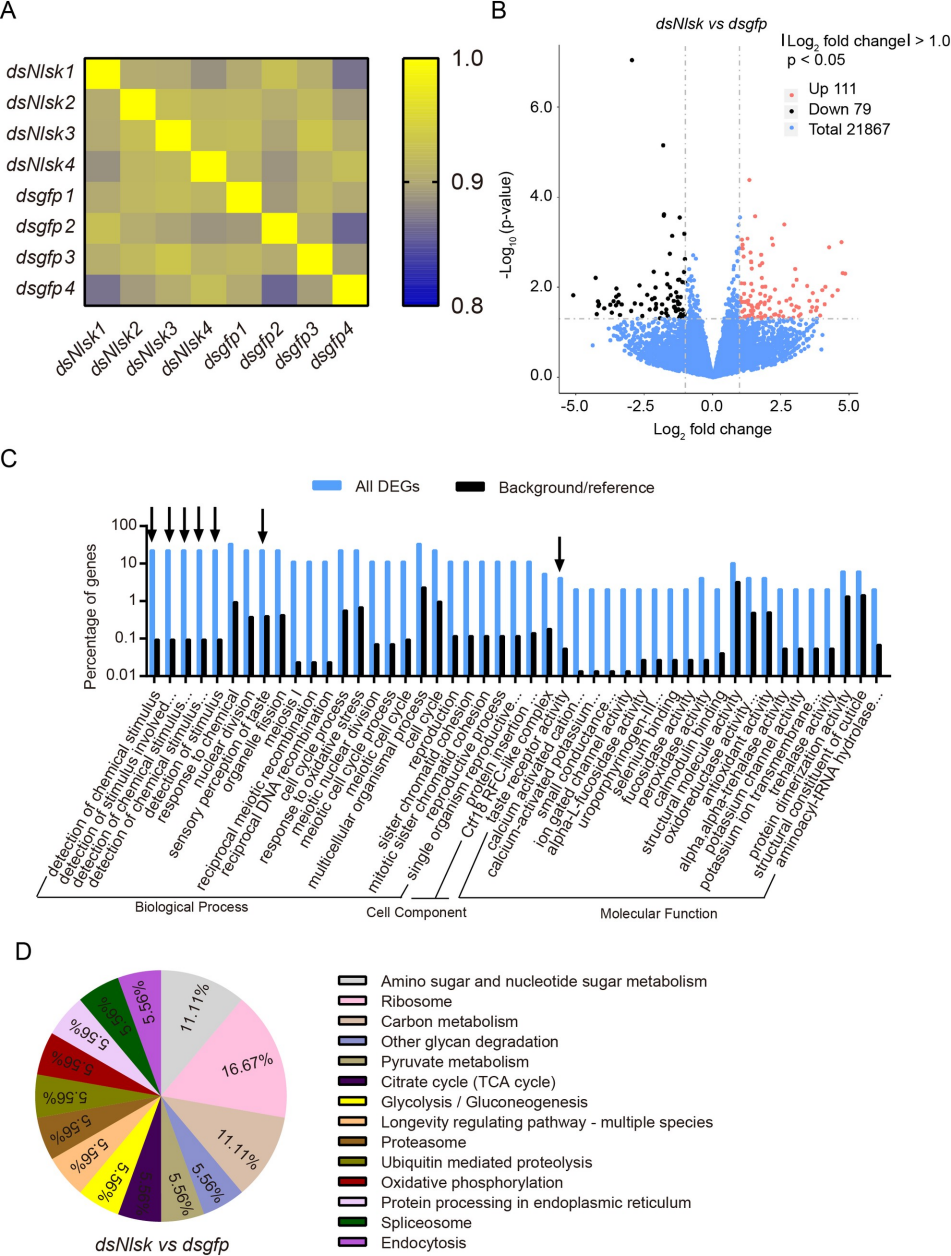
Global gene expression profile in the rice planthopper after *Nlsk* gene knockdown. (**A**) Heatmap showing the square of correlation value from four biological replicates of *dsNlsk* and *dsgfp* groups analyzed by RNA-seq. The square of correlation value was assessed by using the Pearson correlation. (**B**) Volcano plot of differentially expressed genes comparing the *dsgfp* and *dsNlsk* treated brown planthoppers. The red dots indicate significantly (*p* < 0.05 and > 2-fold) upregulated genes. The black dots indicate significantly (*p* < 0.05 and > 2-fold) downregulated genes. (**C**) WEGO (Web Gene Ontology Annotation Plotting) output for *Nlsk* regulated genes. The histogram shows the percent of genes with GO terms enriched in each category. Arrows point to interesting GO categories affected by *Nlsk* knockdown. Hypergeometric test (FDR-adjusted): black arrow indicated *p* < 0.05. (**D**) The pie chart shows the percentage of regulated transcripts distribution in different pathways/processes identified by KEGG pathway analysis.

The mapped reads were used to quantify the expression profiling by FPKM (fragments per kilobase of transcript per million mapped fragments) method (S4 Table). The correlation among four biological replicates in each treatment was then assessed, in terms of the whole genomic FPKM value by using the Pearson method [59]. The heatmap reveals that the square of correlation value ranged from 0.886 to 0.925 and from 0.861 to 0.916 in the ds*Nlsk*–injected and ds*gfp*-injected control groups, respectively (Fig 2A). To identify differentially expressed genes (DEGs) in response to *Nlsk* silencing, the DEGs were screened according to the Noiseq method, which is effective in controlling the rate of false positives [60]. With a cutoff of |Log_2_ fold change| > 1 and *p* < 0.05, a total of 190 DEGs were identified in *Nlsk*–depleted brown planthoppers, including 111 up-regulated and 79 down-regulated DEGs (Fig 2B and S5 Table).

To obtain a functional classification, these DEGs were assigned to three main GO (Gene Ontology) categories: biological processes (37, 71.42%), cellular components (4, 14.29%), and molecular functions (5, 14.29%) which were further organized into 47 subcategories (Fig 2C and S5 and S6 Tables). Furthermore, GO analysis revealed an enrichment of GO terms related to detection of chemical stimulus, detection of stimulus involved in sensory perception, detection of chemical stimulus involved in sensory perception of taste, detection of stimulus and taste receptor activity (Fig 2C and S6 Table) suggesting that genes involved in these processes are regulated by *Nlsk*. For further categorization, the annotated genes were also mapped to KO (KEGG orthologous) terms in the KEGG database. In total, 50 genes (26.32%) could be accessed with a KO number (S5 Table) and were significantly enriched in several vital physiological processes associated with sugar and carbon metabolism (Fig 2D). For example, we identified upregulation of a gene encoding the putative juvenile hormone (JH) binding protein *takeout* (*Nlto*, Gene ID: 111048647, 111048649, and 111045963), the ortholog of which, in *Drosophila*, plays an important role in circadian activity and feeding behavior [61,62]. Other upregulated genes of interest are associated with sensory perception of sweet taste, including gustatory receptors (Gr) for sugar taste Gr64f-like (*NlGr64f*, 111045484) and Gr43a-like (*NlGr43a*, 111056166) (S4 and S5 Tables) [51,63,64]. Furthermore, genes coding for NADH-cytochrome b5 reductase 2-like (CYB5R2, 111046783), and NADH-cytochrome b5 reductase 4-like (CYB5R4, 111052735), the orthologs of which, in mice, play an important role in sugar metabolism [65], were down-regulated. Meanwhile, the *Nlsk* gene (*Nlsk*, 111047610 and 111060391) as positive indicator of our transcriptome data, was significantly repressed after *Nlsk* gene silencing (S4 and S5 Tables). To validate our RNA-seq data, we performed qRT-PCR on six selected genes, three up-regulated and three down-regulated genes in the *dsNlsk*-injection. The qPCR results are consistent with the RNA-seq data (S1 Fig and S4 Table).

### *Nlsk* positively regulates *takeout* gene expression that inhibits feeding behavior of brown planthopper

**Fig 3:**
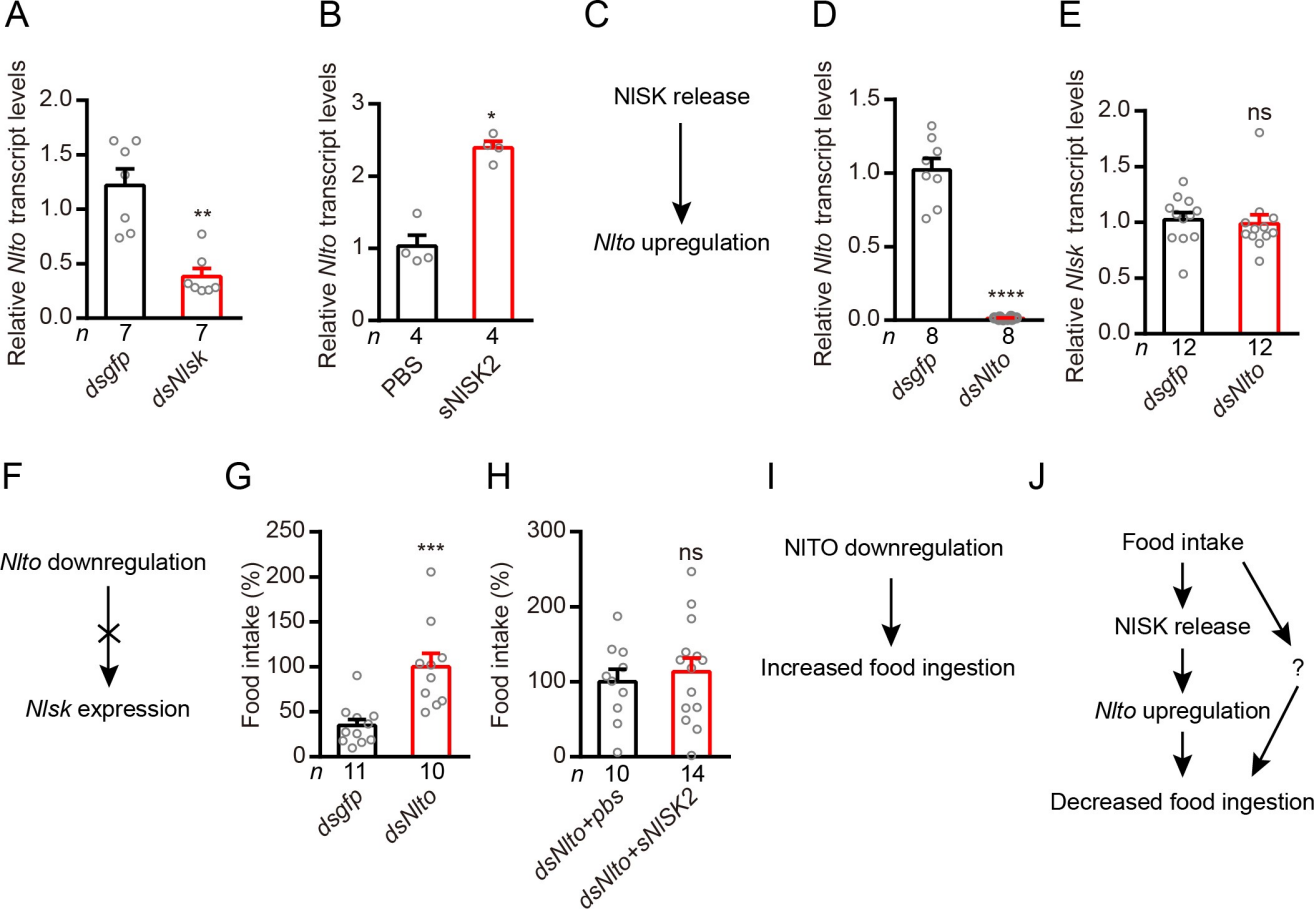
Takeout (to) is a downstream signal of SK that inhibits food intake in the rice planthopper. (**A**) Knockdown of *Nlsk* results in downregulation of *Nlto* gene in the brown planthopper. All data are presented as means ± s.e.m. **p < 0.01; Mann–Whitney test. (**B**) Injection of sNlSK2 increases gene expression level of *Nlto*. *p < 0.05; Mann–Whitney test. (**C**) Model of the NlSK promotes *Nlto* expression. (**D**) Downregulation of *Nlto* gene using *Nlto*-RNAi leads to a reduction in mRNA expression level. ****p < 0.0001; Student’s *t* test. (**E**) Knockdown of *Nlto* have no impacts on expression of *Nlsk* gene in brown planthopper. All data are presented as means ± s.e.m. ns, no significant difference; Mann–Whitney test. (**F**) Model showing that *Nlto* has no effects on *Nlsk* expression. (**G**) Food intake after silencing *Nlto* gene. The *dsNlto*-injected nymphs eat three times more food than *dsgfp*-injected nymphs in the normal conditions. All data are presented as means ± s.e.m. ***p < 0.001; Mann–Whitney test. (**H**) Food intake after silencing the *Nlto* gene with injection of pbs or sNlSK2. No difference was observed between these two conditions. All data are presented as means ± s.e.m. ns, no significant difference; Student’s *t* test. (**I**) Model of the Takeout in the food inhibition. (**J**) Simplified model of the Takeout as a downstream signal of NlSK involved in the feeding inhibition. The question mark indicates one or more possibly additional signals.

*Takeout* (*To*) has been shown to play an important role in the feeding behavior of *Drosophila* [61,62]. To confirm whether *Nlsk* knockdown impairs the expression of *takeout* gene (*Nlto*), we performed qRT-PCR to quantify the expression level of *Nlto* gene from the *dsNlsk*-injected and *dsgfp*-injected brown planthopper. Our results show that knockdown of *Nlsk* expression significantly down-regulates *Nlto* expression (Fig 3A). Furthermore, we found that injection of sNlSK2 significantly induces expression of *Nlto* gene (Fig 3B). These results indicate that NlSK signaling positively regulates the expression of *Nlto* gene (Fig 3C). Next, we asked whether down-regulation of *Nlto* gene could affect expression of *Nlsk*. After silencing of *Nlto* gene, we did not observe any change in the expression of the *Nlsk* gene (Fig 3D and 3E). These results indicate that *Nlto* has little impact on expression of *Nlsk* (Fig 3F). Since *to* regulates feeding behavior in *Drosophila* [61,62], we were interested to test its role in the planthopper. Indeed, we observed that brown planthoppers with silenced *Nlto* gene consume more food than the *dsgfp*-injected controls (Fig 3G). Interestingly, sNISK2 injection does not inhibit feeding in animals with simultaneous *Nlto* knockdown (Fig 3H). Hence, NlTO inhibits food ingestion (Fig 3I). Together with previous findings, we propose that feeding induces secretion of NlSK, which promotes release of Takeout and inhibition of feeding behavior in the brown planthopper (Fig 3J). Again, we should point out that it is likely that there are further molecular components involved in regulation of feeding state-dependent food ingestion (Fig 3J).

### *Nlsk* negatively regulates gustatory sugar receptor *Gr64f* expression and thereby reduces feeding in the brown planthopper

**Fig 4:**
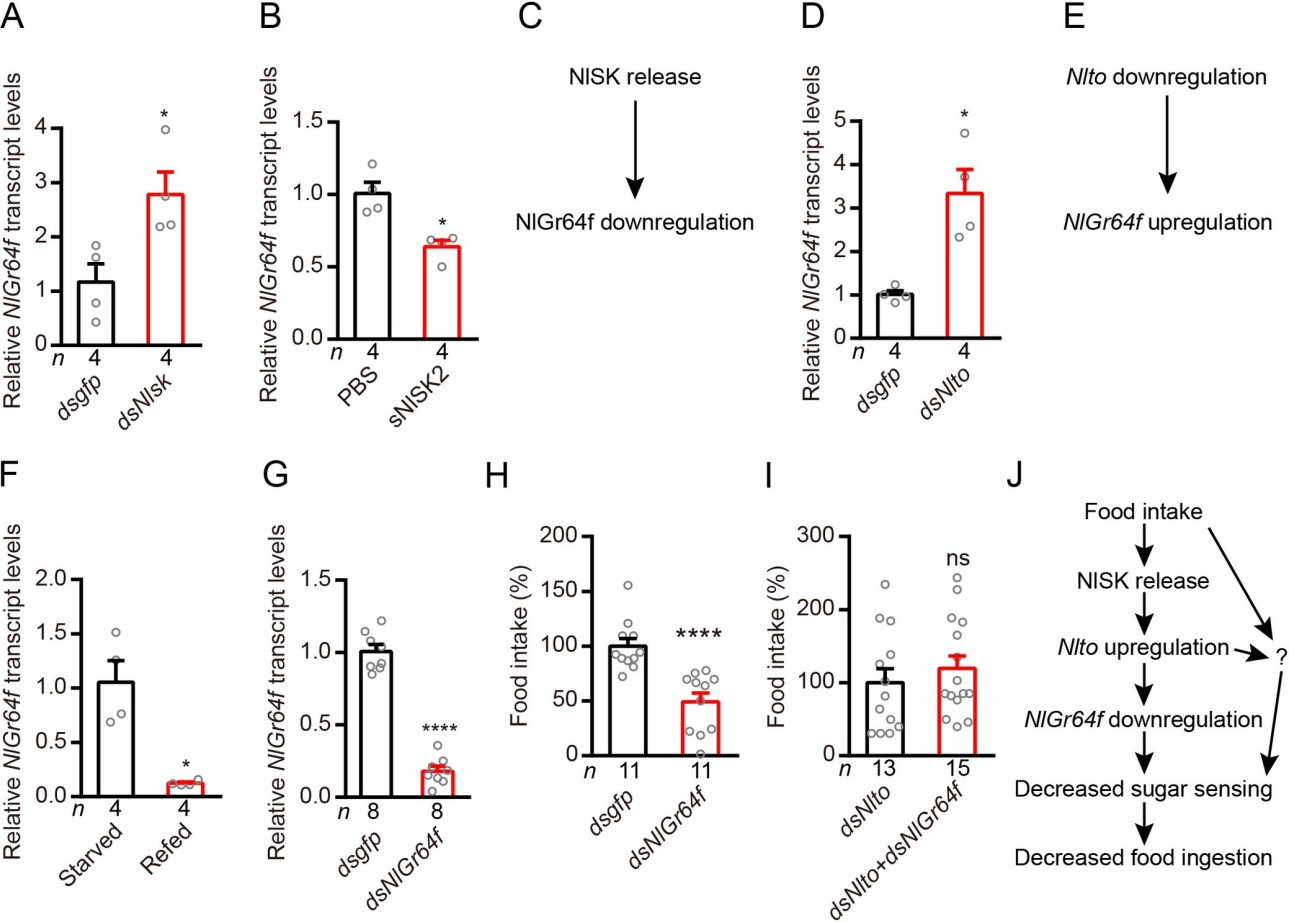
Sulfakinin inhibits expression of the sweet gustatory receptor Gr64f, which promotes food ingestion in the rice planthopper. (**A**) Downregulation of *Nlsk* gene using *Nlsk*-RNAi (*dsNlsk*) leads to up-regulation of transcript of sweet sensing *NlGr64f*. *p < 0.05; Mann–Whitney test. (**B**) Injection of sNlSK2 leads to down-regulation of *NlGr64f* gene. *p < 0.05; Mann–Whitney test. (**C**) Model showing that NlSK inhibits *NlGr64f* expression. (**D**) Downregulation of *Nlto* gene using *Nlto*-RNAi (*dsNlto*) leads to up-regulation of transcript of sweet sensing *NlGr64f*. *p < 0.05; Mann–Whitney test. (**E**) Model showing that *Nlto* inhibits *NlGr64f* expression. (**F**) Refeeding for 5 hr after 24 hr starvation decreases *NlGr64f* transcript. *p < 0.05; Mann–Whitney test. (**G**) Downregulation of *NlGr64f* gene using *NlGr64f*-RNAi (*dsNlGr64f*) leads to a reduction in mRNA expression level. ****p < 0.0001; Student’s *t* test. (**H**) Downregulation of *NlGr64f* gene decreases the food intake of brown planthopper. ****p < 0.0001; Mann–Whitney test. (**I**) Double knockdown of *Nlto* and NlGr64f does not rescue the increased food consumption seen with *Nlto* knockdown alone. All data are presented as means ± s.e.m. ns, no significant difference; Student’s *t* test. (**J**) Simplified model showing that the feeding state regulates SK signaling which in turn modulates *Nlto* and *NlGr64f* signaling (sweet sensing) and thereby feeding. The question mark indicates one or more possibly additional signals.

Interestingly, we found that silencing *Nlsk* gene leads to an up-regulation of gene transcription of the sugar-sensing gustatory receptor *Gr64f* (Fig 4A). Furthermore, injection of sNlSK2 down-regulates *Gr64f* gene expression (Fig 4B). These results indicate that NlSK signaling negatively regulates expression of *NlGr64f* (Fig 4C). Next, we showed that silencing of *Nlto* also increased the expression of *NlGr64f* (Fig 4D) indicating that *Nlto* inhibits expression of *NlGr64f* (Fig 4E). We found that the expression of *NlGr64f* was also influenced by feeding state and displays a phenotype opposite to that of the *Nlsk* gene (Fig 1B). Thus, refeeding after starvation inhibits the expression of *NlGr64f* (Fig 4F). Then, we asked whether silencing of the *NlGr64f* gene impairs feeding behavior. Down-regulation of the *NlGr64f* gene indeed diminishes food intake compared to control animals (Fig 4G and 4H). Next, we tested whether *NlGr64f* is mediating the effects of *NIto*. The double knockdown of *Nlto* and *NlGr64f* has no significant difference with *Nlto* knockdown alone in the food consumption (Fig 4I). These results suggest that food intake promotes NlSK production and release, which in turn induces *Nlto* expression and inhibits *NlGr64f* expression and leads to decreased food consumption. However, our findings indicate that food intake and *Nlto* might target also other factors, which affect food consumption (Fig 4J). We also found that *Nlsk* manipulations alter the expression of another sweet-sensing gustatory receptor, *NlGr43a*. Injection of *dsNlsk* increased NlGr43a expression, whereas injection of sNlSK2 decreased its expression (S2A and S2B Fig). However, we focused on Gr64f in the following analysis of *Drosophila*.

### Drosulfakinins (DSKs) signal satiety in *Drosophila*

**Fig 5:**
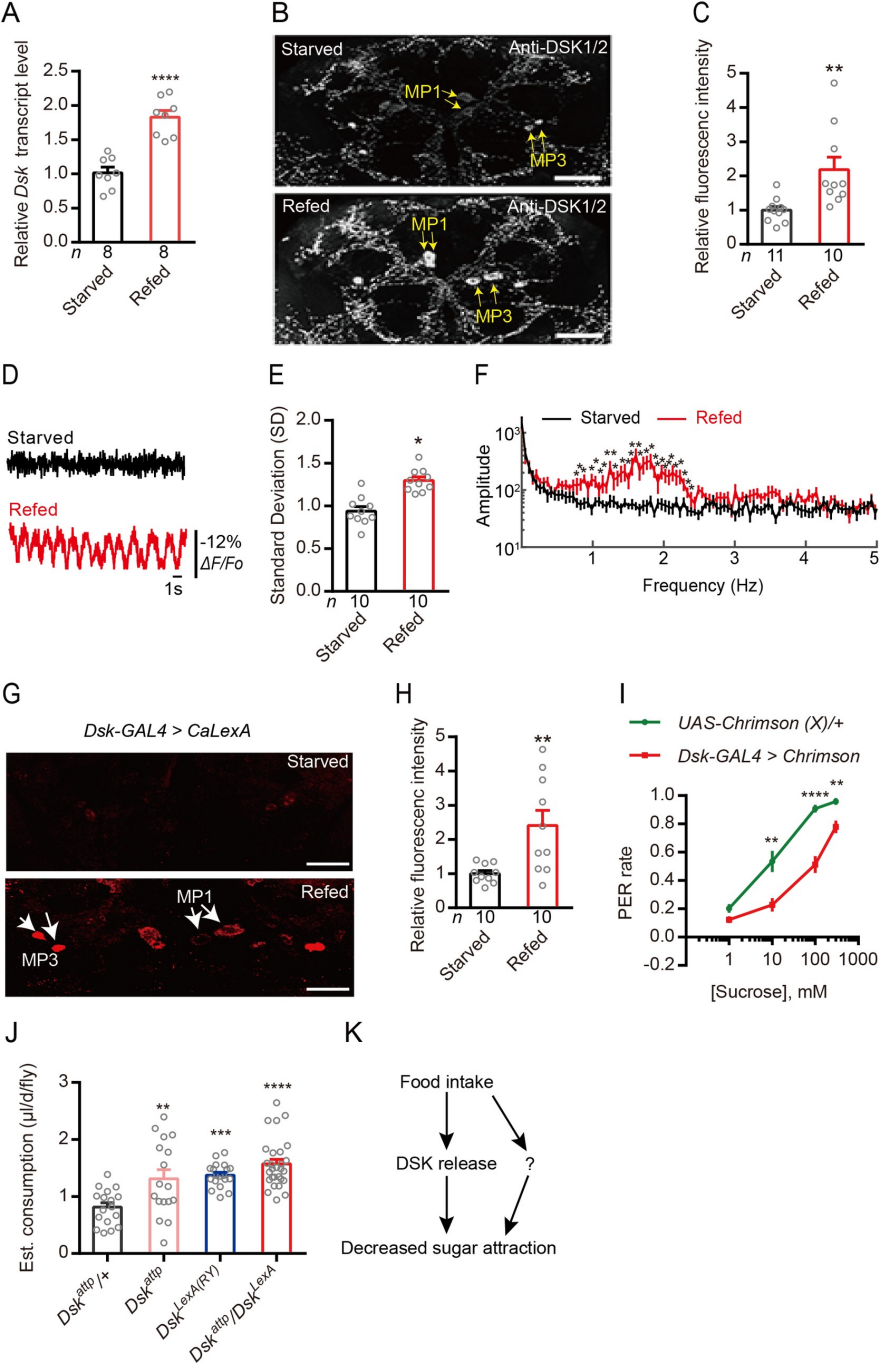
Drosulfakinin (DSK) signals satiety and inhibits feeding in *Drosophila*. (**A**) Refeeding after 24h starvation increases *Dsk* transcript in the head of *Drosophila*. ****p < 0.0001; Student’s *t* test. (**B and C**) Refeeding after 24h starvation increases DSK peptide expression as revealed by anti-DSK1/2 immunolabeling. Yellow arrows indicated the MP1 and MP3 neurons. Scale bars, 50 μm. **p < 0.01; Student’s *t* test. (**D**) Representative optical recordings of spontaneous membrane activity in median protocerebrum (MP1) neurons (Somata) in the starved (0.5% agar for 24 hours) or in the refed (fed 1.5 h after 24 h starvation in 0.5% agar) of flies that Dsk-GAL4 drive the voltage indicator *ArcLight* maintained in 12 hr:12 hr light:dark conditions. (**E**) Standard deviations (SDs) over the recording trial were computed for each field. Refed SD is significantly greater than starved. *p < 0.05; Student’s *t* test. (**F**) Power spectrum was computed for each terminal field using fast Fourier transform with 0.05 Hz bin width. Re-fed power is significantly greater than starved power between 1 Hz to 2.5 Hz. n = 14 for starved and n = 30 for refed. *p < 0.05, **p < 0.01; Student’s *t* test. (**G**) Representative images showing *CaLexA* signals in DSK expressing MP1 neurons of flies exposed to starvation and refeeding. Starved: flies were raised in 0.5% agar for 24 hours; Refed: flies were raised in fly food 1.5 h after 24 h starvation in 0.5% agar. Red: maximal intensity of *CaLexA* signals. Scale bar, 50 μm. (**H**) Quantification of the signal intensity of *CaLexA* signals in DSK expressing MP neurons from flies treated with conditions shown in (G). **p < 0.01; Student’s *t* test. (**I**) Optogenetic activation (19 μm/mm^2^) of *Dsk-GAL4* neurons expressing *Chrimson* is sufficient to inhibit proboscis responses. A fly immobilized in a pipet tip was stimulated on the labellum with different concentrations of sucrose in the presence of light stimulus. We used two values to score the PER assay. A score of 1.0 indicates a fly that extended its proboscis and ingested after being presented with the probe. The score was 0 if the fly failed to extend its proboscis. n = 10 trials. **p < 0.01, ****p < 0.0001; Mann–Whitney test. (**J**) Three Dsk mutants show increased food consumption compared to wildtype in the CAFE essay. **p<0.01, ***p<0.001, ****p<0.0001, Kruskal–Wallis test followed by Dunn’s multiple comparisons test. (**K**) Simplified model showing that DSK inhibits feeding. Note that this model and those in subsequent figures with *Drosophila* data are highly simplified and serve to summarize our data, rather that presenting complete and accurate signaling pathways. The question mark indicates one or more possibly additional signals.

Next, we used the genetic model insect, *Drosophila melanogaster*, to further investigate mechanisms of DSK signaling in modulation of gustatory sugar reception and feeding. Earlier studies have implicated DSK signaling in regulation of food intake [33,37,66,67]. First, we found that the *Dsk* mRNA level is significantly higher in heads of flies refed after 24 h starvation, compared to those from starved flies (Fig 5A). Consistent with this, immunohistochemistry showed that the level of DSK peptides (anti-DSK1/2) in refed flies is higher than that in starved ones (Fig 5B and 5C). These results are similar to the findings in the brown planthopper (Fig 1D and 1E). To monitor activity in Dsk expressing neurons related to feeding, we next expressed the genetically encoded fluorescent voltage indicator *ArcLight* [68] in *Dsk-GAL4* neurons. We observed a higher level of spontaneous activity in the Dsk-expressing medial protocerebrum (MP1) neurons [41] in refed flies compared to that in starved flies (Fig 5D–5F). We ruled out the possibility that this change in activity was induced by altered expression level of the *GAL4* RNA that was under control by the *Dsk* gene promoter, since we found that the *GAL4* expression in the Dsk-GAL4/+ fly was not influenced by feeding state (S3 Fig). Furthermore, we utilised an activity reporter system, calcium-dependent nuclear import of LexA (CaLexA) [69], to reveal whether increased activity of the Dsk-expressing MP1 neurons correlates with refeeding after starvation. Our analysis shows that flies refed after starvation exhibit significantly increased Ca^2+^ activity in the MP1 neurons, compared to starved flies (Fig 5G and 5H). To further confirm our findings, we employed a red-shifted channel rhodopsin, *CsChrimson* [70] as an optogenetic effector under control of the Dsk-GAL4. After light activation of Dsk-GAL4 expressing neurons, we found that the PER (proboscis extension response) of flies was significantly decreased compared with control flies (Fig 5I). However, thermal activation of DSK neurons expressing UAS-dTrpA1 under *Dsk*-GAL4 control did not inhibit feeding behavior (S4 Fig). One reason may be that the long-term activation ultimately reduces the signal from the DSK neurons and/or that the sustained activation desensitizes the response to DSK in target neurons. We also found that *Dsk* mutants display increased motivation to feed in the capillary feeding (CAFE) assay [71] (Fig 5J). Similar results were also previously reported [66]. However, silencing of the *Dsk* gene using Dsk-GAL4 has no significant effect on feeding behavior in *Drosophila* as shown in PER and CAFE experiments (S5A and S5B Fig). It might be that in our hand RNAi is not sufficient induce a behavior phenotype. Another possibility is that Dsk is not the only satiety signal which regulates sweet sensing or feed behavior. These results suggest that re-feeding after starvation increases DSK expression and activates *Dsk*-expressing MP neurons, which release DSK to inhibit feeding behavior (Fig 5K). Our data (S4 and S5 Figs) also suggest that food intake might decrease sugar attraction through additional pathways (see Fig 5K).

### The sugar receptor Gr64f promotes motivation to feed in *Drosophila*

**Fig 6:**
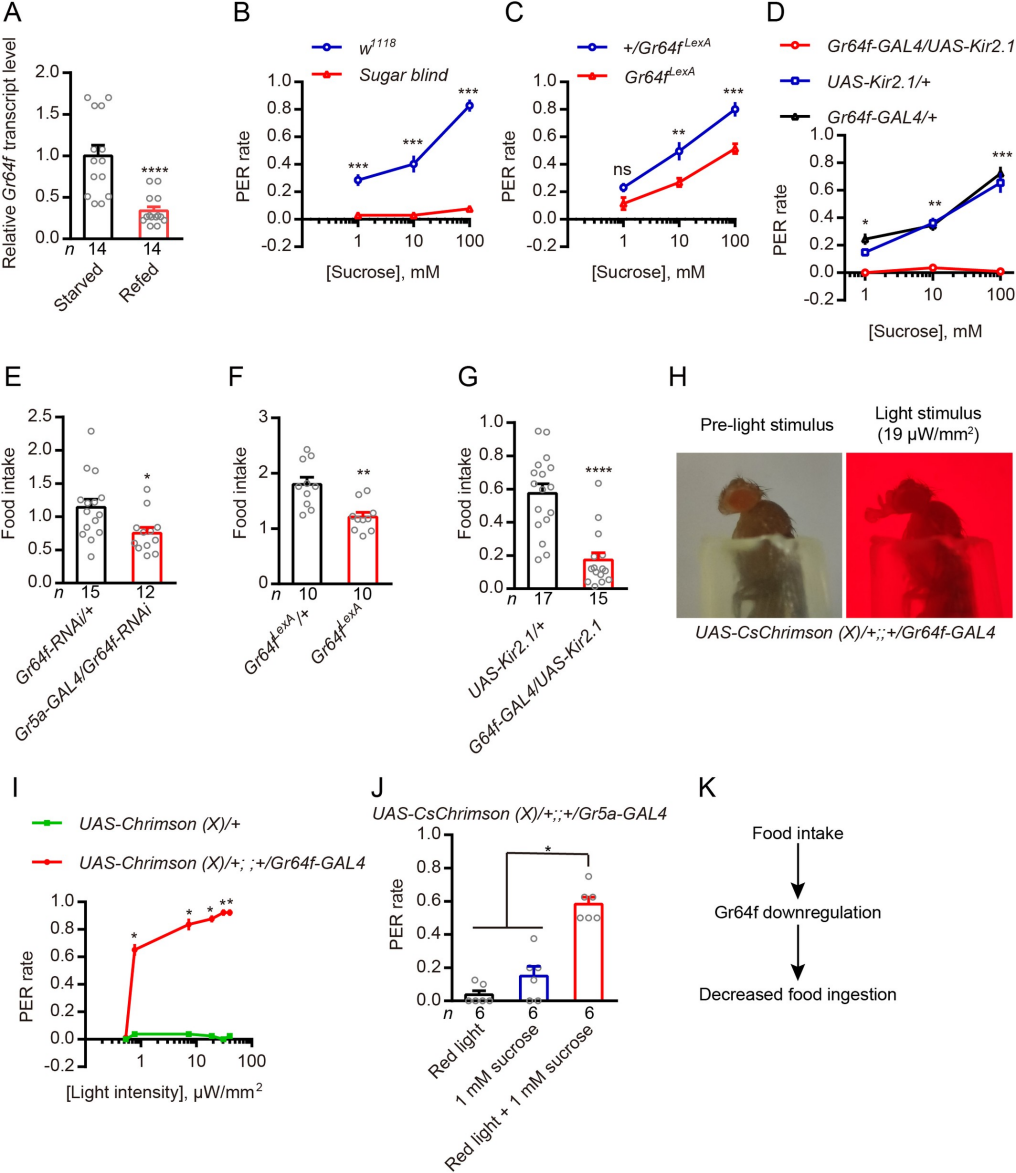
In *Drosophila*, Gr64f promotes feeding. (**A**) Refeeding after 24h starvation decreases *Gr64f* transcript. ****p < 0.0001; Student’s *t* test. (**B**) The sugar blind mutant (all eight known sugar receptors mutated) show almost no motivation to feed in proboscis extension reflex (PER). n = 10 trials. ***p < 0.001; Mann–Whitney test. (**C**) Gr64f mutant (*Gr64f*^*LexA*^) flies show less motivation to feed in PER. n = 10 trials. ***p < 0.001, **p < 0.01, ns, no significant; Mann–Whitney test. (**D**) Silencing *Gr64f-GAL4* neurons by expressing the hyperpolarizing channel Kir2.1 also caused loss of motivation to feed in PER. n = 10 trials. ***p < 0.001, **p < 0.01, *p < 0.05; Kruskal–Wallis test followed by Dunn’s multiple comparisons test. (**E**) Silencing the *Gr64f* gene using the Gr5a-GAL4 driver leads to decreased food consumption compared to wildtype flies in the CAFE essay. *p < 0.05; Student’s *t* test. Food intake is the estimated food consumption (μl) of one female per day. (**F**) *Gr64f* mutant (*Gr64f*^*LexA*^) flies show decreased food consumption compared to wildtypes in the CAFE essay. **p < 0.01; Student’s *t* test. (**G**) Silencing *Gr64f-GAL4* neurons by expressing the hyperpolarizing channel Kir2.1 also leads to decreased food consumption in the CAFE assay. ****p < 0.0001; Mann–Whitney test. (**H**) Dynamics of light-induced proboscis extension after photoactivation in a fly expressing *Gr64f-Gal4*, *UAS-CsChrimson*. (**I**) Relationship between the intensity of the stimulating light and PER rate using the indicated flies. A fly immobilized in a pipet tip was stimulated with red light at the indicated intensity and the light-induced proboscis extension of the fly was recorded. We used two values to score the PER assay. A score of 1.0 indicates a fly that extended its proboscis after being presented with the light. The score was 0 if the fly failed to extend its proboscis. n = 8 trials. **p < 0.01, *p < 0.05; Mann–Whitney test. (**J**) Increasing the baseline activity of sweet GRNs (*UAS-Chrimson (X)/+;; +/Gr5a-GAL4*) by photo-activation with 0.78 μW/mm^2^ of red light increases the probability that the fly shows PER upon sugar stimulation (1 mM sucrose) compared with either red light stimulation (0.78 μW/mm^2^) or 1 mM sucrose alone. *p < 0.05; Kruskal–Wallis test followed by Dunn’s multiple comparisons test. (**K**) Simplified model showing that Gr64f promotes feeding.

We next tested whether sugar-sensing GRNs promote feeding behavior in *Drosophila*. First, we found that the level of *Gr64f* transcription was down-regulated in the refed flies compared with starved ones (Fig 6A), which is opposite to the *Dsk* transcription (Fig 5A). Then, we utilized mutants that lacked all eight known sugar receptors (*sugar blind*) or the critical co-receptor Gr64f (*Gr64f*^*LexA*^) in tests of sugar sensing [72,73] by PER analysis. As expected, sugar blind or *Gr64f*^*LexA*^ flies show almost no, or lower, responses to sucrose solutions (Fig 6B and 6C), similar to flies with their sweet GRNs selectively silenced (Fig 6D). We also find that Gr64f mutants or flies with *Gr64f* gene knockdown in the Gr5a-GAL4 expressing neurons consume less food than controls (Fig 6E and 6F). This is similar to flies with their sweet GRNs selectively silenced by expressing a hyperpolarizing potassium channel (Fig 6G). Next, we tested whether activation of sweet neurons is sufficient to induce feeding behavior. We expressed the optogenetic effector *CsChrimson* under control of the *Gr64f-GAL4*. As with the preceding results, control animals display a low rate of PERs upon stimulation with light stimuli. However, the *UAS-CsChrimson (X)/+;;+/Gr64f-GAL4* animals show a prominent dose-dependent PER responses under light stimuli (Fig 6H and 6I and S1 Movie). Similar results were also reported in previous work [74,75]. Furthermore, we found that increasing the baseline activity of sugar GRNs by opto-genetical activation increases the probability that the fly shows PER upon sugar stimulation (Fig 6J). These results indicate that starvation promotes Gr64f expression and activates sugar-sensing GRNs, reflecting a starved state that promotes feeding behavior (Fig 6K).

### DSK and takeout negatively regulate sugar receptor *Gr64f* expression in *Drosophila*

**Fig 7:**
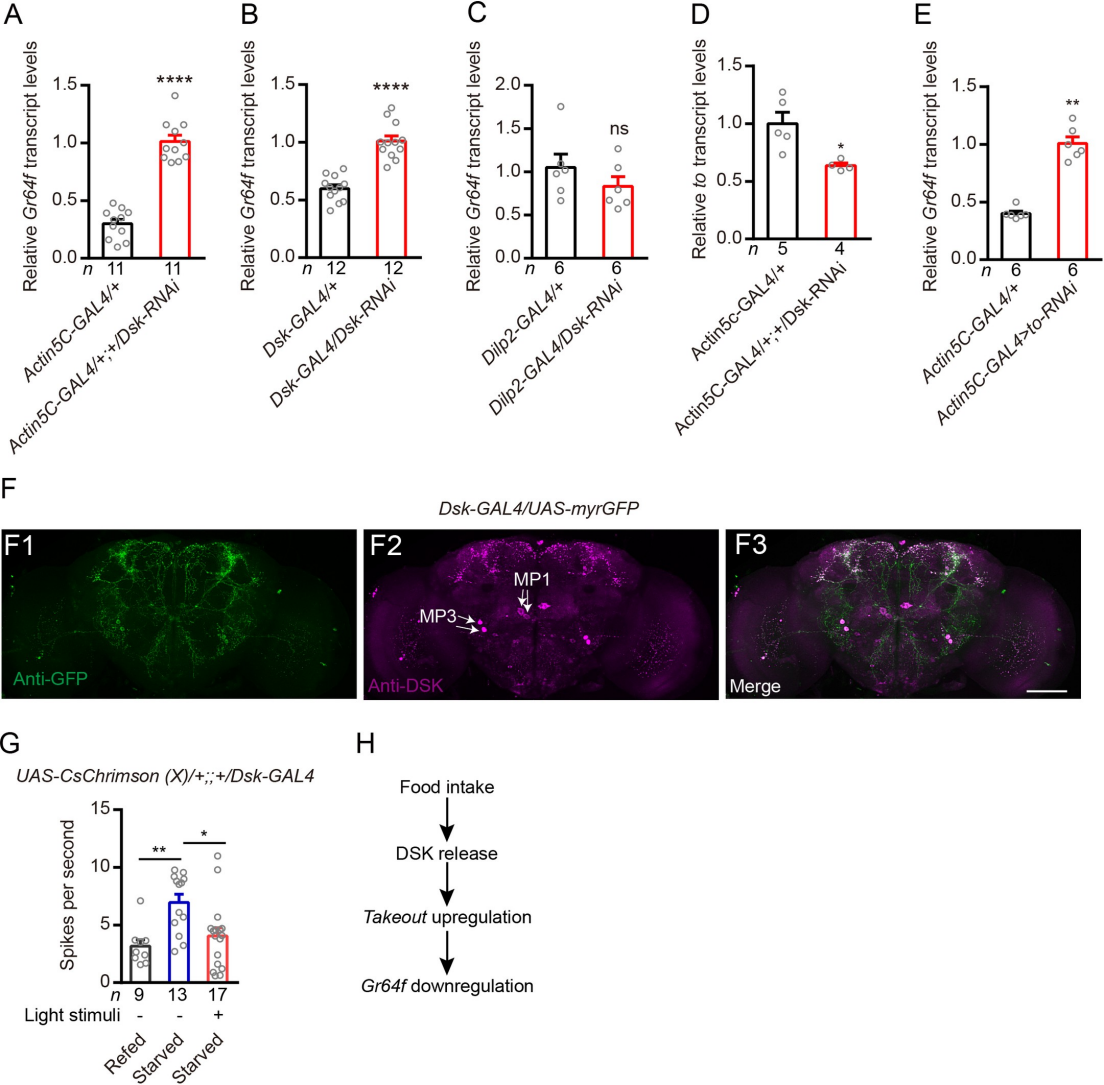
In *Drosophila* DSK and TO inhibits Gr64f gene transcription and optogenetic activation of Dsk-GAL4 neurons decreases the sensitivity of gustatory neurons in starved flies. (**A and B**) Relative expression levels of *Gr64f* transcripts in DSK deficient files. Depletion of *Dsk*, either globally in all cells (*Actin5C-GAL4*) or in the Dsk-GAL4-expressing cells increased *Gr64f* transcripts in starved flies. ****p < 0.0001; Student’s *t* test. (**C**) Silencing only *Dsk* in the IPCs with RNAi (*Dilp2-GAL4/Dsk-RNAi*; red bars) did not affect *Gr64f* transcript levels. ns: not significant; Mann–Whitney test. (**D**) Relative expression levels of *to* transcripts in *Dsk* deficient files. Depletion of *Dsk* in all cells (*Actin5C-GAL4*) decreased *to* transcripts in flies. *p < 0.05; Mann–Whitney test. (**E**) Relative expression levels of *Gr64f* transcripts in TO deficient files. Depletion of *to* in all cells (*Actin5C-GAL4*) increased *Gr64f* transcripts in flies. **p < 0.01; Mann–Whitney test. (**F**) Expression pattern of *Dsk*-GAL4 in the brain revealed by anti-GFP (F1) and anti-DSK (F2). Scale bars, 50 μm. (**G**) Electrophysiological recordings reveal that starved flies (24 h) show increased sensitivity of GRNs compared with refed flies (fed 1.5 h after starvation) and optogenetic activation of *Dsk*-GAL4 neurons (30 min) decreases the sensitivity of gustatory neurons in starved flies (24 h). **p < 0.001; *p < 0.05; Kruskal–Wallis test followed by Dunn’s multiple comparisons test. (**H**) Simplified model of the feeding state, DSK, TO and Gr64f interaction.

To test whether silencing *Dsk* gene expression affects the expression of *Gr64f* also in *Drosophila*, we used a UAS-Dsk-RNAi transgene targeted either globally, to Dsk-expressing neurons, or to insulin-producing cells (IPCs) [41]. Knockdown with the global GAL4 driver (Actin5C-GAL4), and the specific driver (Dsk-GAL4), resulted in up-regulation of the *Gr64f* gene (Fig 7A and 7B). However, diminishing of *Dsk* in the insulin producing cells (IPCs) using Dilp2-GAL4 (S6 Fig) has no significant effect on *Gr64f* gene expression (Fig 7C). Our previous studies showed that Dsk-GAL4 is expressed in two sets of MP neurons and a subset of the IPC neurons [33,41], and our data here indicate that DSK from MP neurons, but not from IPCs is responsible for affecting *Gr64f* expression. Similar to brown planthopper, in *Drosophila*, silencing of *Dsk* gene negatively regulates *takeout* (*to)* expression (Fig 7D). Thus, we asked whether *to* also negatively regulates Gr64f expression. Indeed, our results show that reducing *to* expression using an Actin5C-GAL4 driver upregulates *Gr64f* expression (Fig 7E).

### SK is not likely to be the only satiety signal that mediates nutritional state dependent reduction of sweet attraction

Next, we asked whether SK is the only satiety-induced neuropeptide that modulates sweet sensation. We found that starvation renders wild type flies more sensitive to sucrose than re-fed flies (S7A Fig). However, *sugar blind*, which is the sugar receptor mutant, displayed no difference of PER rate between starved and re-fed conditions (S7B Fig). Similar results were also observed when we silenced sweet GRNs by expressing the Kir2.1 channel under Gr64f-GAL4 control (S7C Fig). These findings are expected since we eliminated the sugar receptors or silenced the sweet-sensing GRNs. However, *dsk* mutant flies and flies with silencing of *Dsk*-GAL4 expressing neurons exhibited responses similar to wild type flies where starved flies are more sensitive than re-fed flies (S7D and S7E Fig). These results indicate that SK is not the only signal that contributes to a reduction of sweet attraction in re-fed flies.

### Optogenetic stimulation of Dsk-GAL4 neurons decreases the sensitivity of gustatory neurons in starved flies

Next, we set out to identify the potential mechanism by which DSK neurons modulate Gr64f-expressing sugar-sensing GRNs. We found that sugar-sensing GRNs in the proboscis and Dsk-expressing neurons in the brain project their axons to overlapping areas in the subesophageal zone (SEZ) of the brain (S8A and S8B Fig). We did not detect Dsk-expressing neurons in the proboscis or proleg tarsi (S8C and S8D Fig). Our Dsk-GAL4 line labels eight MP neurons in the fly brain (Fig 7F), but, only MP1 neurons project to SEZ of the brain [41]. Next, we asked whether activation of Dsk-GAL4 neurons could impair the sensitivity of GRNs. As previous studies reported [62], we also observed that starved flies show increased sensitivity of GRNs compared with re-fed flies (Fig 7G). However, activation of Dsk-GAL4 neurons for 30 min decreases the sensitivity of GRNs in starved flies (Fig 7G). These results indicate that re-feeding induces release of DSK, which promotes TO and then inhibits sugar receptor expression and sugar-sensing neuron activity (Fig 7H).

### Distribution of sulfakinin receptor (SKR) in the sweet-sensing gustatory neurons

**Fig 8:**
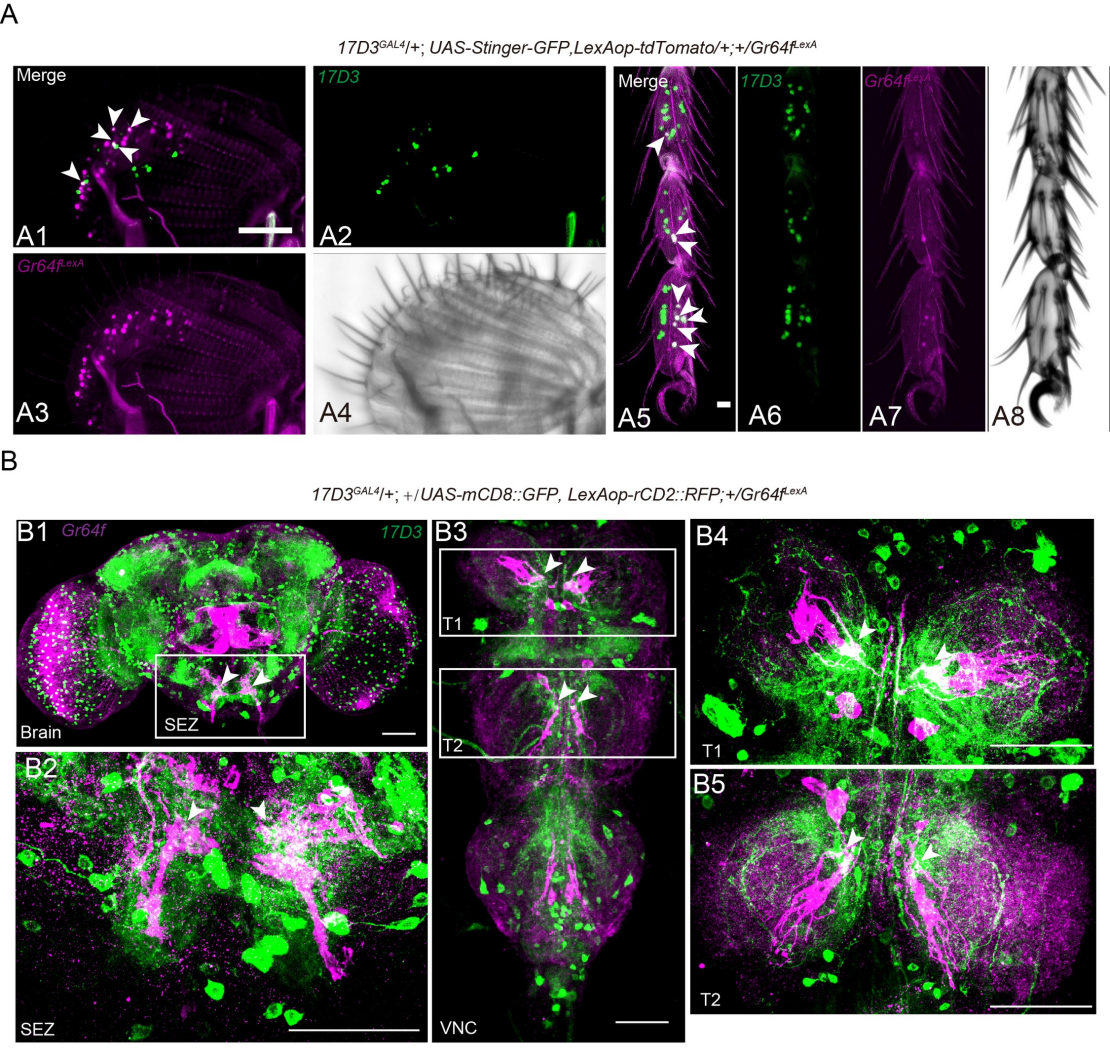
In *Drosophila* the DSK receptor, CCKLR-17D3, is expressed in *Gr64f*^*LexA*^ expressing neurons. (**A**) *17D3*^*GAL4*^, *UAS-Stinger-GFP* (green) superimposes with *Gr64f*^*LexA*^, *LexAop > tdTomato* (magenta) expressing cells in the labellum (A1-A4) and proleg tarsi (A5-A8) of flies. Overlap is in white. The white arrowheads (A1 and A5) indicate the positive neural cells co-labeled by *17D3*^*GAL4*^ and *Gr64*^*LexA*^. Scale bar: 50 μm. (**B**) Double labeling of 17D3^GAL4^-expressing neurons and Gr64f^LexA^-expressing sweet neurons in the brain (B1), SEZ (B2), and VNC (B3-B5). SEZ: subesophageal zone; VNC: ventral nerve cord; T1-T3: thoracic ganglion 1–3. Scale bar: 50 μm.

Earlier studies have shown that GRNs in *Drosophila* are modulated by neuropeptides to adjust sensitivities to sweet and bitter taste [11]. Hence, in hungry flies NPF, via dopaminergic cells, increases sweet sensitivity and sNPF decreases bitter sensitivity [11]. Thus, we asked whether DSK receptors are expressed in the sweet-sensing GRNs of the gustatory system. Two G-protein coupled receptors, the CCK-like receptors (CCKLR)-17D1 and CCKLR-17D3, have been identified as the receptors of DSK peptides in *Drosophila* [76–78]. As we failed to generate functional CCKLR-17D1 and CCKLR-17D3 antibodies, we produced a knock-in GAL4 into the start codon of each of CCKLR-17D1 and CCKLR-17D3 [41] for expression analysis (S9A Fig). We did not detect expression of *17D1*^*GAL4*^ in cells of the leg tarsi, maxillary palps or proboscis (S9B Fig). However, we found that 17D3^GAL4^ drives expression broadly in cells of the proleg tarsi, proboscis and maxillary palps (S10 Fig). Next, we asked whether the CCKLR-17D3 receptor is co-expressed with sugar receptors in the gustatory receptor neurons (GRNs). Double labeling of *Gr64f*^*LexA*^ and *17D3*^*GAL4*^ expression (*17D3*^*GAL4*^*; LexAop-RedStinger*, *UAS-stinger-GFP/+; +/Gr64f*^*LexA*^) revealed that five pairs of neurons of the labellum and eight of each proleg tarsi are co-labeled by *17D3*^*GAL4*^ and *Gr64f*^*LexA*^ (Fig 8A). Thus, we have evidence that CCKLR-17D3, one of the two known sulfakinin receptors, is expressed in some of the Gr64f-expressing neurons in the proboscis and leg tarsi (Fig 8A). We also show here that *Gr64f*^*LexA*^ and *17D3*^*GAL4*^ labeled neurons of the proboscis extend their axons to overlapping areas in the SEZ of the brain (Fig 8B1 and 8B2). In addition, we also detected their axons in overlapping areas of the prothoracic neuromere of the ventral nerve cord (Fig 8B3–8B5).

### CCKLR-17D3 signaling in GRNs mediates feeding-induced modulation of PER

**Fig 9:**
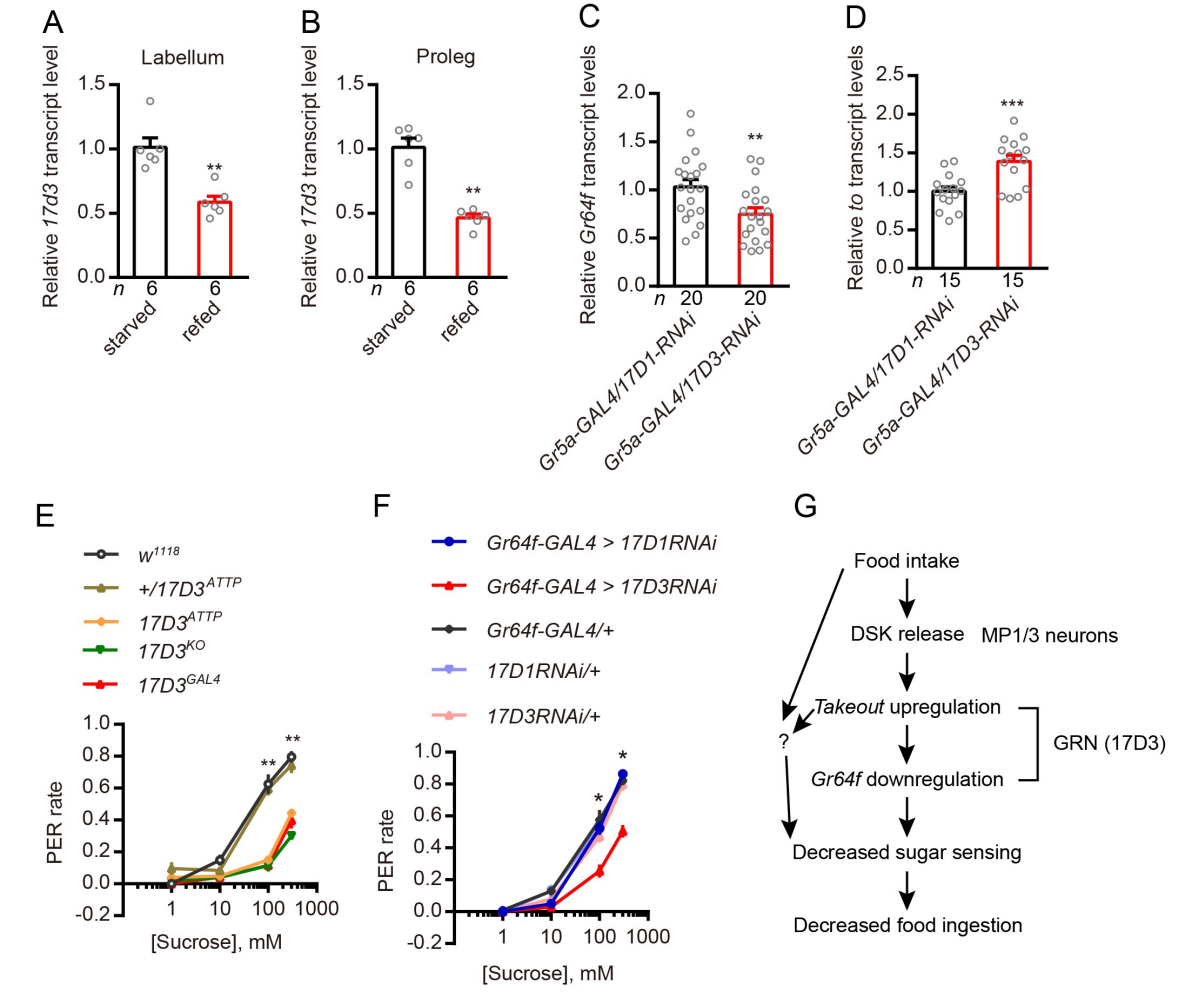
In *Drosophila* activity of the DSK receptor (CCKLR-17D3) is necessary for PER. (**A and B**) Refeeding after 24h starvation decreases *17d3* mRNA transcript in the labellum and proleg tarsi. All data are presented as means ± s.e.m. **p < 0.01; Mann–Whitney test. (**C and D**) Silencing of the *17d3* gene in sweet GRNs leads to downregulation of *Gr64f* and upregulation of the *to* gene. All data are presented as means ± s.e.m. **p < 0.01, ***p < 0.001; Student’s *t* test. (**E**) 17D3 mutants show decreased motivation to feed in PER. **p < 0.01; Kruskal–Wallis test followed by Dunn’s multiple comparisons test. (**F**) Silencing of *17D3* gene in *Gr64f-GAL4*-expressing neurons showed decreased motivation to feed in PER. *p < 0.05; Kruskal–Wallis test followed by Dunn’s multiple comparisons test. (**G**) Simplified model of SK as satiety signal that reflects internal states and inhibits sweet sensation (an external stimulus). Note that this model is simplified and does not exclude additional parallel signaling pathways that modulate gustation and feeding. The question mark indicates one or more possibly additional signals.

Next, we asked whether the inhibition of the PER by DSK that we observed is mediated by its receptor, CCLR-17D3, which is expressed by the sweet-sensing GRNs. To address this, we first found that the level of *ccklr-17d3* transcription in the labellum and proleg is down-regulated in the refed flies compared to starved flies (Fig 9A and 9B), which is similar to the transcription of Gr64f (Fig 6A). Secondly, to exclude that DSK might have an indirect effect by acting on 17D3 receptors in other cells, we specifically silenced the two DSK receptors in Gr5a neurons. Only knockdown *17d3* gene expression in Gr5a neurons leads to the downregulation of *Gr64f* and an upregulation of *to* (Fig 9C and 9D). Thirdly, we found that 17D3 mutant flies exhibit decreased PER compared to control flies (Fig 9E). In contrast, 17D1 mutants do not display a significant difference to controls in our PER assay (S9C Fig). Furthermore, we knocked down either of the DSK receptors (CCKLR-17D1 and CCKLR-17D3) by crossing each of *UAS-17D1-RNAi* and *UAS-17D3-RNAi* with *Gr64f-GAL4*. We measured the PER in our behavioral assay and found that flies lacking 17D3 in sweet-sensing GRNs exhibit a significantly decreased PER, whereas 17D1 knockdown had no effect (Fig 9F). Thus, 17D3 expression in Gr64f expressing GRNs mediates the starvation-dependent enhancement of PER. Importantly, it should be noted that our data suggest that the 17D3 receptor mediates an inhibitory signal in the Gr64f expressing GRNs, and possibly other brain neurons. Thus, the DSK signal after food intake inhibits activity in Gr64f expressing GRNs, which reduces sweet sensing, and consequently knockdown of 17D3 leads to diminishment of this inhibition and thereby a decrease in PER. Other studies have also indicated inhibitory action of SK receptors in specific neurons. For instance, Wu et al. [41] found that 17D3 activation leads to an inhibition of activity in specific fruitless expressing neurons (P1) resulting in a suppression of sexual arousal and Wicher et al. [56] showed that SK, as a satiety signal, hyperpolarizes dorsal unpaired median neurons and thus affects general activity in a cockroach.

## Discussion

Food seeking and feeding are under complex control by neuronal circuits as well as by neuropeptides and peptide hormones [3,12,15,40,55,79–81]. Thus, sensory systems, central circuits and interorgan communication, in a nutrient-dependent fashion, contribute to feeding decisions and regulation of food ingestion. In this study, we have analyzed mechanisms of nutrient state-dependent peptidergic regulation of gustatory inputs and feeding.

We show herein that in both the planthopper *N*. *lugens* and the fly *Drosophila*, SK signaling mediates satiety and decreases sensitivity of gustatory neurons (GRNs) expressing the gustatory sugar receptor Gr64f. Thus, SK release not only decreases food intake [33,34,82], but also downregulates attraction to sugar. We find that food ingestion diminishes and starvation increases Gr64f expression under control of SK signaling. In the brown planthopper, the gene *takeout* is upregulated by SK, and we showed that knockdown of *takeout* upregulates *Gr64f* and increases feeding in both insects.

We performed additional experiments in *Drosophila* to reveal further SK signaling mechanisms. Calcium and membrane activity in DSK expressing MP neurons in the brain increase after feeding, suggesting that these neurons receive nutrient signals. We could also show that optogenetic activation of DSK neurons in the brain decreases the PER, whereas activation of *Gr64f* expressing GRNs increases the PER. DSK neurons were found to make functional contacts with Gr64f expressing GRNs in the SEZ, and one of the two DSK receptors, CCKLR-17D3, is expressed in Gr64f expressing GRNs in proleg tarsi, labellum and maxillary palps. Furthermore, the *ccklr-17D3* levels are downregulated in these appendages after feeding. Our data suggest that the CCKLR-17D3 inhibits activity in Gr64f expressing GRNs and hence knockdown of this receptor leads to a decrease in sugar sensitivity and reduced PER. In *Drosophila*, *takeout* knockdown not only increases feeding, but also expression of Gr64f, further suggesting a role of *takeout* in DSK-mediated satiety signaling. Hence, we find that food ingestion activates SK signaling in the two insects and that SK acts to decrease expression of a sweet receptor and thereby diminish food attraction and feeding (Fig 9G). However, our data also suggest that SK in not the only satiety-induced signal that contributes to nutritional state modulation of sugar gustation (see Fig 9G). As mentioned in the introduction, neuropeptides such as NPF and sNPF, as well as dopamine, are also known to regulate gustatory perception [11]. Furthermore, food search also depends on olfaction, and neuropeptides like MIP, CCH2amide and TK have been found to modulate the sensitivity of ORNs [6,10,21–24]. We can also not rule out the possibility that manipulating DSK signaling could affect food intake and dietary status and thus indirectly influence sweet sensing via other signaling pathways.

Invertebrate SKs and the vertebrate CCKs are known as satiety inducing peptides that regulate food ingestion [34,36,38,44–46,81,83,84]. However, these peptides also act in a multitude of other regulatory functions both centrally and in the periphery [37,38,85]. In insects SKs play additional roles in gut motility [42], digestive enzyme production and release [35,47,86], regulation of sexual arousal [41], as well as hyperactivity and aggression [39,67]. Similarly, in mammals CCK stimulates pancreatic enzyme secretion and release of insulin and glucagon, gallbladder contraction and gut motility, and is implicated in fear, anxiety, and aggression [see [36,38,85,87]]. In addition CCK has diverse roles in the brain as a neuromodulator regulating other neurotransmitter systems [see [85]]. Thus, in planthoppers and flies, one can expect that SK acts at several levels and that these actions are coordinated to generate a relevant behavioral and physiological outcome.

In *Drosophila* and planthopper there are cell bodies of SK-producing neurons only in the brain, and to our knowledge no SK peptide is produced in the intestine or other tissues [41,58]; see also FlyAtlas2 http://flyatlas.gla.ac.uk, [88]. We show here that four pairs of posterior DSK interneurons, MP1 and MP3, with wide arborizations in the brain are likely to underlie the regulation of *Gr64f*-expressing GRNs. These MP1 neurons display increased calcium and spontaneous electric activity after feeding, and we demonstrate here that the MP1 neurons have processes that superimpose GRN axon terminations in the SEZ [see also [41]]. Furthermore, optogenetic activation of DSK neurons rapidly inhibits the PER suggesting direct neuronal connections. A subset of the brain insulin-producing cells (IPCs) is also known to co-express DSK [33,39,41], but our experiments exclude these cells in modulation of GRNs. The IPCs may instead release DSK as a circulating hormone to act on the intestine in regulation of digestive enzymes as demonstrated in the planthopper and other insects [see [35,47,86]], but not yet shown in *Drosophila*.

We found that knockdown of *takeout* increases feeding, and also that this upregulates expression of *Gr64f* in planthopper and fly. Interestingly, it has been shown earlier that *takeout* is expressed in GRNs of the labellum in *Drosophila* and that mutant flies are deficient in sugar sensing and regulation of food ingestion [62]. *Takeout* mutants are also aberrant in their starvation-induced locomotor activity and display increased mortality during starvation [61,62]. Furthermore, the gene *takeout* is expressed also in the intestine and fat body, is under control by the circadian clock, and was proposed to link circadian rhythms and feeding behavior [61]. Since *takeout* encodes a putative juvenile hormone (JH) binding protein, it was suggested that it regulates levels of circulating JH and that this may impart the effects on locomotor activity and food intake, as well as effects on metabolism [62]. The role of *takeout* in the GRNs in modulation of sugar sensitivity by regulating *Gr64f* in the same cells requires further investigation.

As mentioned, SK acts at several levels of the CNS and periphery and modulates conflicting behaviors such as food search, feeding, sexual arousal and aggression [33,39,41,67]. These studies show that *Dsk* neurons increase aggression and decrease feeding and mating. The DSK-expressing MP neurons are central in suppression of both feeding (this study), mating [41] and aggression [66], but aggression was also found dependent on DSK in the IPCs [39,67]. Modulation of feeding and mating relies on different downstream circuits. For suppression of male sexual behavior DSK (from MP neurons) acts on male-specific fruitless-expressing P1 neurons that coordinate arousal-related behaviors such as sex, sleep and locomotion [41]. In parallel, as we show here, the same DSK neurons target GRNs to suppress sweet attraction and inhibit feeding. It is not clear at present how DSK released from IPCs suppresses feeding and what the hormonal DSK targets are [33].

Our data herein suggest that SK is not the only signal that modulates sweet attraction and feeding behavior in response to food ingestion. Other systems, such as the four widely arborizing SIFamide expressing neurons of the pars Intercerebralis, are also known to coordinate hunger and satiety signals to stimulate appetitive behavior and suppress mating behavior and sleep [18,89]. Also this SIFamide system acts at different levels, such as olfactory and gustatory circuits, as well as sleep and activity circuits and fruitless expressing neurons [18,90]. Several studies have also shown peptidergic modulation of ORNs and GRNs to promote state-dependent food seeking in *Drosophila*. Thus, nutrient dependent insulin signaling modulates sNPF and TK signaling to alter sensitivity of ORNs [6,10,21], whereas NPF and sNPF regulate sweet and bitter sensitivity, respectively [11]. NPF and sNPF are also known to act in other circuits to modulate feeding, metabolism and sleep [see e. g. [25–28,30–32]]. Previous studies found that short-term starvation changes dopamine signaling, which leads to sensitization of sweet sensing neurons [11]. However, in our experiments the starvation time is 24 hours, which is longer than in the earlier experiments. Thus, we assume that in our experiments the effect on the sugar response is not mediated by dopamine.

In summary, we show that DSK secreted from brain neurons regulates sugar sensitivity of DSK receptor-expressing GRNs in response to food ingestion and thereby diminishes food attraction (Fig 9G). Mechanistically this state-dependent desensitization of specific GRNs is by SK receptor mediated downregulation of Gr64f expression in GRNs, possibly involving action of *takeout*. Importantly, the mechanisms described herein are conserved in *Drosophila* and the brown planthopper, although these insects are only distantly related. It is likely that our findings have revealed only a portion of the mechanisms involved in mediating nutritional state dependent regulation of sensory perception and subsequent feeding behavior.

## Materials and methods

### Experimental insects and husbandry

The brown planthopper *N*. *lugens* was reared on ‘Taichung Native 1’ (TN1) rice (*Oryza sativa* L.) seedlings in the laboratory and maintained at 27 ± 1 ^∘^C, with 70 ± 10% relative humidity, under a 16 h: 8 h light dark photoperiod [91].

Flies were maintained on standard molasses/cornmeal/yeast/agar food at 25°C on a 12:12 LD cycle with humidity set to 60 ± 5% unless otherwise indicated. The following fly strains were ordered from Bloomington Stock Center: *w*^*1118*^ (Bloomington Stock number: #5905); *Canton-S* (#64349); *Gr64f-GAL4* (#57668; [92]); *Actin5c-GAL4* (#4414); *UAS-ArcLight* (#51056; [68]); *UAS-RedStinger* (#8547; [93]); *UAS-CaLexA* (#66542; [69]); *UAS-Stinger-GFP* (#84277); *UAS-Chrimson*, *attp18* (#55134; [70]); *UAS-Chrimson*, *attp40* (#55135; [70]); *UAS-dTrpA1* [94]; *UAS-NaChBac* (#9467; [95]); *LexAop-rCD2*::*RFP*, *UAS-mCD8*::*GFP* (#67093); *UAS-nSyb-spGFP*^*1-10*^, *LexAOP-CD4-spGFP*^*11*^ (#64314; [96]); *LexAOP-nSyb-spGFP*^*1-10*^, *UAS-CD4-spGFP*^*11*^ (#64315; [96]); *Δ17D1[Df(1)Exel9051]* (#7762;*17D1*^*KO*^ [78]). *UAS-DskRNAi* (THU2073), *UAS-17D1RNAi* (THU2656), *UAS-17D3RNAi* (THU2970), and *UAS-Gr64fRNAi* (TH04838.N) were purchased from Tsinghua Fly Center at the Tsinghua University [97,98]. *UAS-Kir2*.*1* [99], *LexAop2-IVS-nlstdTomato* [93], *Dsk-GAL4*, *Dsk-LexA*, *17D3*^*KO*^, *17D3*^*GAL4*^, *Dsk*^*1*^, and *UAS-mCD8*::*GFP* have been described previously [41]. *Dilp2-GAL4* (#37516; [100]) was kindly provided by Dr. Wei Song. *Gr64f*^*LexA*^ and *sugar blind* flies (*R1*,*Gr5a*^*LexA*^*;+; Δ61a*, *Δ64a-f*) were a gift from Dr. Hubert Amrein [72,73]. *Dsk*^*attP*^, *Dsk*^*LexA(RY)*^, *17D1*^*attP*^, and *17D3*^*attP*^ were kind gifts from Dr. Yi Rao [101].

### Peptide synthesis

Sulfakinin peptides were synthesized by Genscript (Nanjing, China) Co., Ltd. Peptides mass was confirmed by MS and the amount of peptide was quantified by amino acid analysis. The amino acid sequence of the peptides used in this study are: *N*. *lugens* sulfakinin 1: (NlSK1): SDDYGHMRFamide; sulfakinin 2: (NlSK2): GEADDKFDDYGHMRFamide; sulfated sulfakinin1 (sNlSK): SDDY(SO_3_H)GHMRFamide; sulfated sulfakinin2 (sNlSK2): GEADDKFDDY(SO_3_H)GHMRFamide.

### Gene cloning and sequence analysis

We used the NCBI database with BLAST programs to carry out sequence alignment and analysis. Then we predicted Open Reading Frames (ORFs) with EditSeq. The primers were designed by Primer designing tool–NCBI. Total RNA Extraction was using the TRIzol reagent (Invitrogen, Carlsbad, CA, USA) according to the manufacturer’s instructions. The cDNA template used for cloning was synthesized using the Biotech M-MLV reverse transcription kit and the synthesized cDNA template was stored at -20°C.

The transmembrane segments and topology of proteins were predicted by TMHMM v2.0 (http://www.cbs.dtu.dk/services/TMHMM-2.0/) [102]. Multiple alignments of the complete amino acid sequences were performed with Clustal Omega (http://www.ebi.ac.uk/Tools/msa/clustalo). Phylogenetic tree was constructed using MEGA 7.0 software with the Maximum Likelihood Method and bootstrapped with 1000 replications [103].

### Quantitative RT-PCR

The first-strand cDNA was synthesized with HiScript II Q RT SuperMix for qPCR (+gDNA wiper) kit (Vazyme, Nanjing, China) using an oligo(dT)18 primer and 500 ng total RNA template in a 10 μl reaction, following the instructions. Real-time qPCRs in the various samples used the UltraSYBR Mixture (with ROX) Kit (CWBIO, Beijing, China). The PCR was performed in 20 μl reaction including 4 μl of 10-fold diluted cDNA, 1μl of each primer (10 μM), 10 μl 2 × UltraSYBR Mixture, and 6 μl RNase-free water. The PCR conditions used were as follows: initial incubation at 95°C for 10 min, followed by 40 cycles of 95°C for 10 s and 60°C for 45 s. *N*. *lugens* 18S rRNA or Drosophila *rp49* were used as an internal control (S1 Table). Relative quantification was performed via the comparative 2^−△△CT^ method [104]. We used *Drosophila* heads to analyze the expression of *Dsk* and GAL4 in starved or refed condition.

### RNA interference in *N*. *lugens*

For lab-synthesized dsRNA, *gfp*, *Nlsk*, *Nlto* and *NlGr64f* fragments were amplified by PCR using specific primers conjugated with the T7 RNA polymerase promoter (primers listed in S1 Table). dsRNA was synthesized by the MEGAscript T7 transcription kit (Ambion, Austin, TX, USA) according to the manufacturer’s instructions. Finally, the quality and size of the dsRNA products were verified by 1% agarose gel electrophoresis and the Nanodrop 1000 spectrophotometer and kept at -70°C until use.

The 4th instar nymph of brown planthoppers was used for injection of 60 nl of 5 μg/μl dsRNA per insect. Injection of an equal volume of *dsgfp* was used as negative control. RNAi efficiency was examined by qPCR using a pool of ten individuals on the 3^rd^ day after dsRNA injections. The insects of the third day after dsRNA injection were used for feeding assay and gene relative expression analysis.

### Feeding assay of *N*. *lugens*

The animals were food deprived for 5 h before onset of the experiment to ensure that all experimental animals were in the same nutritional state prior to the experiment. This was based on several periods of starvation (2, 5, 12, and 24 h) we tested, from which a starvation for 5 h gave the best results in terms of intraexperiment variation. The animals were starved in the morning and the experiments were done in the afternoon. The feeding assay method and artificial diet was adopted as previously reported with modification [105]. Briefly, the antifeedant potency of sulfakinins was measured in fourth instar nymphs of *N*. *lugens*. Prior to injection, the peptides were dissolved in PBS. Individual brown planthoppers were then injected with 40 nl of peptide solution (2.25 pmol/insect) or 40 nl of PBS in the lateral side of the abdomen using a FemtoJet system (Eppendorf-Nethler-Hinz, Hamburg, Germany). Immediately after injection, ten animals were placed in separate plastic containers (9 cm long and 2 cm in diameter), provided with 200 μl of artificial diet and allowed to feed *ad libitum* for 24 hours.

For studying the effect of gene silencing on the feeding behavior of *N*. *lugens*, the dsRNA-injected 4^th^ instar nymphs were reared on rice seedlings in the laboratory and maintained at 27 ± 1 ^∘^C, with 70 ± 10% relative humidity, under a 16 h: 8 h light dark photoperiod to recover for 2 d. After 5 hours starvation, ten nymphs were transferred into separate plastic containers (9 cm long and 2 cm in diameter), provided with 200 μl of artificial diet and allowed to feed *ad libitum* for 24 hours as mentioned above. The feeding amount of brown planthopper in each feeding chamber was recorded after 24h. The experiment was repeated at least four times.

### RNA-seq analysis

Total RNA of thirty 4^th^ instar nymphs was isolated at day three after *dsNlsk* or *dsgfp* injections in the 4^th^ instar nymphs using a TRIzol reagent (Invitrogen) according to the manufacturer’s protocol. Library construction and sequencing was performed by Novogene with Illumina HiSeq2000 platform (Novogene Bioinformatics Technology Co.Ltd, Beijing, China). Raw sequence data were submitted to the Short Read Archive (SRA) database of NCBI under the accession numbers SRR12460889 (*dsNlsk-1*), SRR12460895 (*dsNlsk-2*), SRR12460894 (*dsNlsk-3*), SRR12460893 (*dsNlsk-4*) and SRR12460896 (*dsgfp-1*), SRR12460892 (*dsgfp-2*), SRR12460891 (*dsgfp-3*), and SRR12460890 (*dsgfp4*).

The raw data were analyzed after filtering the low-quality sequences. Sequences were aligned to the *Nilaparvata lugens* genome (https://www.ncbi.nlm.nih.gov/genome/?term=Nilaparvata+lugens) using Hisat2 v2.0.5. The expression level of genes from the RNA sequencing was normalized by the FPKM method (Fragments Per Kilobase of transcript sequence per Millions base pairs sequenced). This method considers the effect of sequencing depth and gene length for the reads count at the same time and is currently the most commonly used method for estimating gene expression levels. Differential expression analysis was performed using the DESeq2 R package (1.16.1). The clusterProfiler R package was used for Gene Ontology (GO) enrichment analysis and KEGG pathway analysis [106]. FDR-adjusted multiple tests were added to the hypergeometric test.

### Generation of *17D1*^*GAL4*^ knock-in line

To prepare the 17D1^GAL4^ line, we used CRISPR-HDR (clustered regularly interspaced short palindromic repeats–homology directed repair) method based on previous methods [41]. We chose the upstream and a downstream guide RNAs targeting the part of first exon using the CRISPR Optimal Target Finder: http://tools.flycrispr.molbio.wisc.edu/targetFinder/. In brief, the part of first CCKLR-17D1 coding exon was replaced by GAL4::p65 (S6A Fig). Firstly, two gRNAs (gRNA1: 5′-GATTTATAAACTCGGGTCGCA-3′; gRNA2: 5′-TCACCGACAGCGGAGATCTC-3′) against CCKLR-17D1 were inserted into pCFD4 as previously described [107]. We then fused GAL4::p65 into pHD-DsRed (Addgene #51434) between the EcoRI and the NdeI sites. Next, each homologous arm was subcloned into the pHD-DsRed vector too. We injected the modified pCFD4 and pHD-DsRed plasmids into the embryo of *vas-Cas9* flies (# 51324). The correct insertion was confirmed by 3xP3-DsRed screening and recombination accuracy was confirmed by sequencing.

### NSK/DSK antibody

Rabbit anti-DSK antibody was generated by using the peptide N′-FDDYGHMRFC-C′ that corresponds to the predicted DSK-1 and DSK-2 peptides as antigen as previously reported [41]. We used the DSK antibody to recognize both SK peptides of *N*. *lugens* and *Drosophila* since the antigen share the same sequence in two species.

### Immunohistochemistry

Unless otherwise stated, fourth instar nymph of brown planthopper and 3-5-day old mated female flies were dissected under phosphate-buffered saline (PBS; pH 7.4) or Schneider’s insect medium (S2) as previously described [41,108]. The tissues were fixed in 4% paraformaldehyde in PBS for 30 min at room temperature. After extensive washing with PAT (0.5% Triton X-100, 0.5% bovine serum albumin in PBS), the tissues were incubated in primary antibody for 24 h at 4°C and in secondary antibody for 24 h at 4°C. Primary antibodies used: mouse anti-GFP (Sigma-Aldrich Cat# G6539, 1:1000), rabbit anti-RFP (Abcam Cat#ab62341, 1:1000), mouse anti-Bruchpilot (Developmental Studies Hybridoma Bank nc82, 1:30), rabbit anti-DSK (see antibody generation section, 1:100). Secondary antibodies used: donkey anti-mouse IgG conjugated to Alexa 488 (1:500) or Alexa 555 (1:500) and donkey anti-rabbit IgG conjugated to Alexa 488 (1:500) or Alexa 555 (1:500) (Molecular Probes). The samples were mounted in Vectorshield (Vector Laboratory). Images were acquired with Zeiss LSM 700 confocal microscopes, and were processed with Image J software [109].

To quantify NlSK level in the brain, we stained the brains with DSK antibody (Fig 1E and 1F). The *dsgfp*- or *dsNlsk*-injected samples were processed in parallel and using the same solution and imaged with the same laser power and scanning settings. With the imaged data, we got “Sum Slices” Z-projection of the sub-stacks encompassing whole brain to measure fluorescence intensity (*F*_*dsgfp*_ and *F*_*dsNlsk*_), then select a small region without signal as the background fluorescence (*B*_*dsgfp*_ and *B*_*dsgfp*_) using ImageJ. Then we obtained the relative fluorescence of NlSK as the ratio of NlSK signal to the control signal ((*F*_*dsNlsk*_*—B*_*dsNlsk*_)/(*F*_*dsgfp*_ *− B*_*dsgfp*_)).

### Arclight imaging

Imaging of freshly dissected brain explants of starved (24 hours) or refed (1.5 hours refed after 24 hours starvation) was performed on a Zeiss 710 NLO Axio Examiner confocal microscope using a water immersion objective (Zeiss, Germany). ArcLight was excited with the 488 nm laser. The objective C-mount image was projected onto the 256 × 256 pixel chip controlled by Zen2010 software (Zeiss Germany). Images were recorded at a frame rate of roughly 80 Hz, and depicted optical traces were spatial averages of intensity of all pixels within the region of interest (ROI, in this study, cell bodies of MP1 neurons), with signals processed as previously reported [68]. Statistical analysis and plotting of the data were performed using Excel and Prism GraphPad.

### CaLexA measurements

Calcium activity of DSK neurons following starvation and refed was measured using the calcium-dependent nuclear import of LexA (CaLexA) reporter system [69]. Female flies (4–7-days old) carrying Dsk-GAL4 and UAS-CaLexA system were collected. They were then divided in two groups: one group was starved for 24 hours in presence of water and another group was refed 1.5 hours after 24 hours starvation. Following this period, the fly brains were quickly dissected and were processed for immunohistochemistry. The GFP fluorescence were quantified as described above.

### CsChrimson activation

For optogenetic stimulation, tester flies were collected within twelve hours after eclosion and transferred into a vial with regular food containing 200 μM all-trans retinal (116–31–4, Sigma-Aldrich). The vials were covered by aluminum foil to protect from light for 3–5 days before PER test. We immobilized flies on a glass slide with back down so that the proboscis was exposed to the upside and stimulated the animal with red (620 nm, 0.03 mW/mm^2^, Vanch Technology, Shanghai, China) light. Unless otherwise noted, light stimulation was presented continuously throughout the observation period. Light intensity was measured by placing an optical power meter (PS-310 V2, Gentec, Canada) nearby the location of glass slide.

### *dTrpA1* activation

Experimental flies were maintained at 22°C, cold anesthetized and loaded into CAFE vial, and allowed to recover for at least 30 min at 22°C. Vials containing flies were then placed at experimental temperatures (30°C) for 24 hours to recording the feeding amount.

### Extracellular tip recording

7–10 days old females were used for electrophysiological recordings. All flies were kept in dark after eclosion and fed with 200 μM all-trans-retinal for 3 to 5 days. Before the assays, flies were transferred to a tube contained a filter paper with 2ml of all-trams-retinal solution (200 μM all-trans-retinal diluted in 2 ml ultrapure water) for 24 hours, and then some groups were refed for 1.5 hour. Electrophysiological recording from *Drosophila* labellar taste sensilla were implemented as previously reported [110]. A reference electrode was inserted into dorsal thorax of fly, and proboscis was fully extended. The recording electrode was approached to the tip of a single L-sensilla on labellum and covered ~ 50% of the total shaft length. Beadle-Ephrussi Ringer solution (B&E) was used as the reference electrode electrolyte, which contained 7.5 g NaCl, 0.35g KCl, and 0.279 g CaCl2∙2H2O in one liter of ultrapure water. Using 30 mM tricholine citrate solution (TCC) solved with 100 mM sucrose as the recording electrode electrolyte. Both reference and recording electrodes were capillary glass (borosilicate glass with filament, BF120-69-15; O.D.: 1.2mm, I.D.: 0.69mm) that was pulled on a P-97 puller (Sutter Instrument Corp). The recording electrode was connected to an amplifier (TastePROBE DTP-02, SYNTECH) and a data acquisition device (Axon Digidata 1550B, Molecular Device) under the control of Axon pCLAMP 10.6 software (Molecular Devices). Data was analyzed with Clampfit 10.7 software.

### Proboscis extension response (PER) assays

We performed the PER assays at about 2:00–5:00 pm (ZT6–ZT10) at room temperature. 3-5-day-old females were used for the assays. Before the assays, flies were starved for 24 hours, and refed for 1.5 hour. We first anesthetized flies with carbon dioxide and stuck them on microscope slides. After one hour recovery, we tested flies with water. Proboscis were touched by a water drop, and if the fly did not extend its proboscis in three seconds, we performed the assays with different concentrations of sucrose. Two values were used in the PER assays. A score of 1 means a fly that extended its proboscis and ingested after being fed the sucrose water drop. If not, the score of that fly is 0. We averaged the scores of 5–10 flies as one replicate.

### Capillary feeding (CAFE) assay

This method was modified from Ja et al. [71]. A vial (9 cm height × 2 cm diameter), filled with 5 ml of 1% agarose to provide water for the flies, was used for this assay. Capillaries (5 μl, VWR International) were exchanged daily with new ones containing fresh food solution (5% sucrose and 5% yeast extract dissolved in water). Typically, 24-h feeding of every fly is shown after 1 or 2 d of habituation in this assay. The amount of consumed food minus evaporation was quantified.

### Statistics

We used the GraphPad Prism 7 software package to generate graphs and statistically analyze data. Data presented in this study were first verified for normal distribution by D’Agostino–Pearson normality test. If normally distributed, Student’s t test was used for pairwise comparisons, and one-way ANOVA was used for comparisons among multiple groups, followed by Tukey’s multiple comparisons. If not normally distributed, Mann–Whitney test was used for pairwise comparisons, and Kruskal–Wallis test was used for comparisons among multiple groups, followed by Dunn’s multiple comparisons. All data are presented as mean ± s.e.m. The sample sizes and statistical tests used for each experiment are stated in the figures or figure legends. The raw data of this project are shown in the S7 Table.

## Supporting information

S1 Fig **qRT-PCR analysis of six selected genes from RNA-seq data after *Nlsk* knockdown by RNAi in the brown planthopper (A-F).** All data are presented as means ± s.e.m. ***p < 0.001, **p < 0.01, *p < 0.05; Mann–Whitney test.
(TIF)

S2 Fig Sulfakinin inhibits expression of the fructose receptor Gr43a in the rice planthopper. (**A**) Downregulation of *Nlsk* gene using Nlsk-RNAi (dsNlsk) leads to up-regulation of transcript of sweet sensing *NlGr43a*. *p < 0.05; Mann–Whitney test. (**B**) Injection of sNlSK2 leads to down-regulation of *NlGr43a* gene. ***p < 0.001; Mann–Whitney test.
(TIF)

S3 Fig Relative expression of GAL4 in Dsk-GAL4/+ genotype fly under starved and refed condition. ns: not significant; Student’s t test.
(TIF)

S4 Fig Thermogenic activation of DSK neurons expressing UAS-TrpA1 under Dsk-GAL4 control did not inhibit feeding. All flies were kept in CAFE tubes for 24 hours at 30°C. ns: not significant; Kruskal–Wallis test followed by Dunn’s multiple comparisons test.
(TIF)

S5 Fig Silencing of *Dsk* gene has no significant effect on feeding behavior in *Drosophila*. (**A**) Silencing the *Dsk* gene using the *Dsk-GAL4* driver has no impact on feeding in the proboscis extension reflex (PER). n = 10 trials. ns: not significant; Mann–Whitney test. (**B**) Silencing the *Dsk* gene using the *Dsk-GAL4* driver has no impact on feeding in the CAFE assay. n = 10 trials. ns: not significant; Mann–Whitney test.
(TIF)

S6 Fig Co-labeling of a subset of insulin-producing cells in the *Drosophila* Pars Intercerebralis (PI) region by *Dilp2-GAL4* (green) and *Dsk-LexA* (magenta). Scale bars: 50 μm.
(TIF)

S7 Fig DSK is not the only satiety-signaling molecule that modulates sweet attraction. (**A**) Starved Canton-S showed more motivation to feed in the PER assay. n = 10 trials. *p < 0.05; Mann–Whitney test. (**B and C**) *Sugar blind* mutants and silencing sweet GRNs by expressing the Kir2.1 channel did not influence responses to starvation and re-feeding in the PER. n = 10 trials. ns, no significant; Mann–Whitney test. (**D and E**) Starved *dsk* mutants and flies with silencing of *Dsk*-GAL4 labeled neurons by expressing the Kir2.1 channel displayed more motivation to feed in PER. n = 10 trials. *p < 0.05; Mann–Whitney test.
(TIF)

S8 Fig **Double labeling of *Dsk-GAL4*-expressing neurons and *Gr64f***^***LexA***^**-expressing sweet gustatory neurons in the *Drosophila* brain** (**A**: anterior and **B**: posterior), proleg (**C**) and proboscis (**D**). No Dsk signal was detected on the proleg and proboscis (**C and D**). Scale bar: 50 μm.
(TIF)

S9 Fig (**A**) Generation of knock-in of GAL4 into the CCKLR-17D1 locus. (**B**) No signal was detected when the *17D1*^*GAL4*^ drives stinger and GFP in the proboscis and leg tarsi. Scale bar: 50 μm. (**C**) 17D1 mutants show no decreased motivation to feed in PER compared with control. **p < 0.01; Kruskal–Wallis test followed by Dunn’s multiple comparisons test.
(TIF)

S10 Fig Expression pattern of 17D3GAL4 visualized by UAS-mCD8::GFP in leg tarsi (A1-A3), proboscis and maxillary palps (B1-B3). Scale bar: 50 μm.
(TIF)

S1 Table The primers used in this study. (DOCX)

S2 Table Summary of sequence assembly after RNA-seq of silence Nlsk gene. (DOCX)

S3 Table Number of reads sequenced and mapped to the genome of silence *Nlsk* gene. (DOCX)

S4 Table Fragments per kilobase of transcript per million mapped fragments of all sequenced gene. (XLSX)

S5 Table Go and KEGG analysis of the differential expressed genes between dsNlsk and dsgfp-injceted 4th instar nymphs. (XLSX)

S6 Table Three categories of GO analysis of the differential expressed genes between *dsNlsk* and *dsgfp*-injected 4th instar nymphs. (XLSX)

S7 Table Raw data of this project. (XLSX)

S1 Movie Optogenetic activation of Gr64f-GAL4 labeled sweet is sufficient to trigger proboscis responses. (MP4)
