# Supplementary figures and images for: Cholecystokinin-like peptide mediates satiety by inhibiting sugar attraction
Source: PLoS Genet. 2021 Aug 16;17(8):e1009724. doi: 10.1371/journal.pgen.1009724 (PMC8366971; doi:10.1371/journal.pgen.1009724)

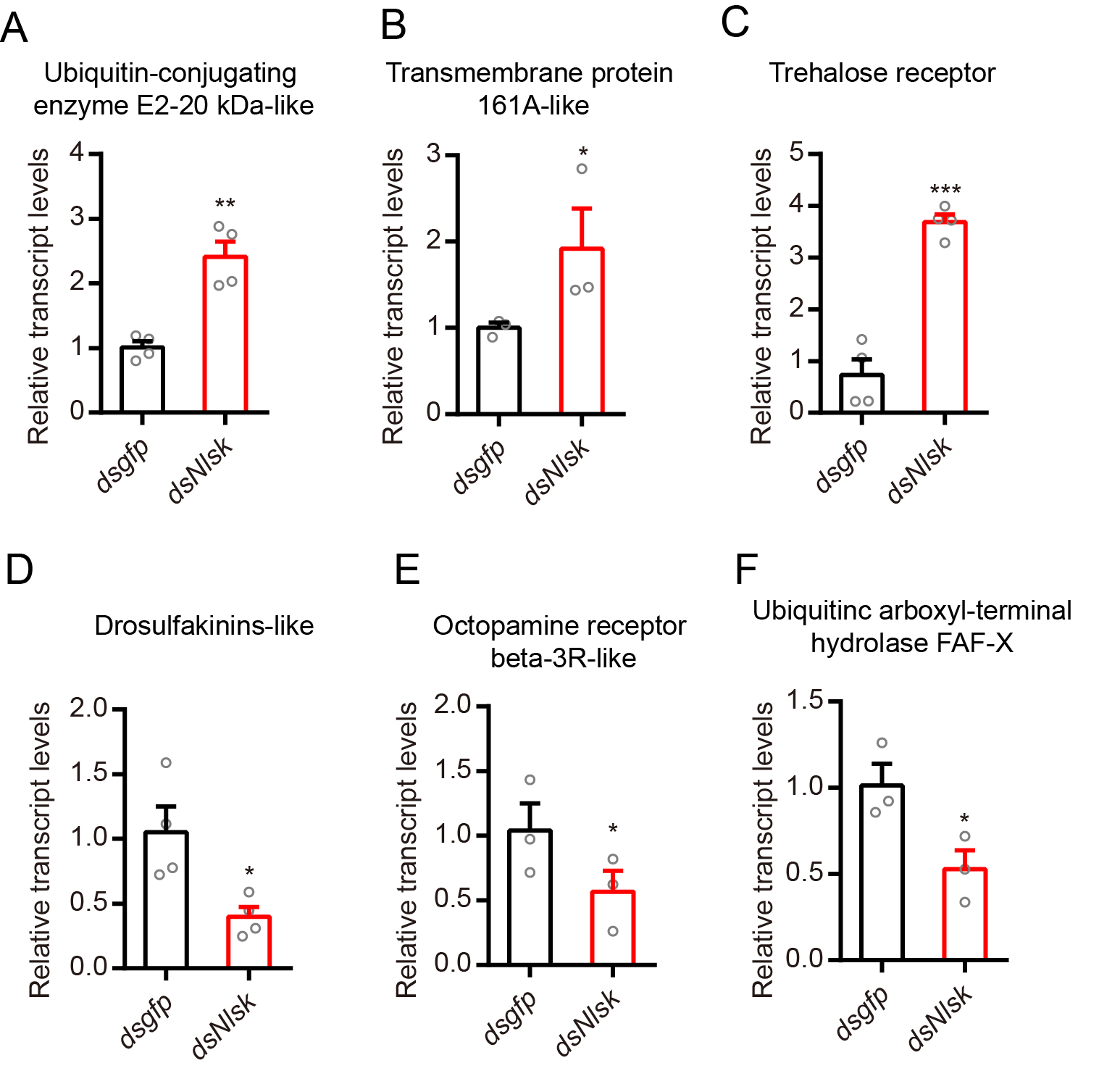

Supplement: S1 Fig — qRT-PCR analysis of six selected genes from RNA-seq data after Nlsk knockdown by RNAi in the brown planthopper (A-F). All data are presented as means ± s.e.m. ***p < 0.001, **p < 0.01, *p < 0.05; Mann–Whitney test. (TIF) [file pgen.1009724.s001.tif]

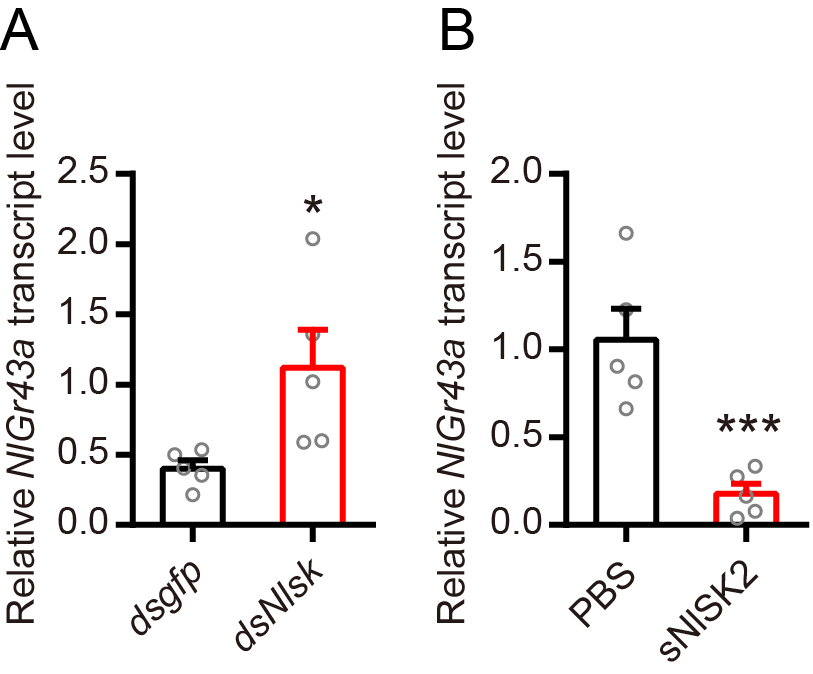

Supplement: S2 Fig — (A) Downregulation of Nlsk gene using Nlsk-RNAi (dsNlsk) leads to up-regulation of transcript of sweet sensing NlGr43a. *p < 0.05; Mann–Whitney test. (B) Injection of sNlSK2 leads to down-regulation of NlGr43a gene. ***p < 0.001; Mann–Whitney test. (TIF) [file pgen.1009724.s002.tif]

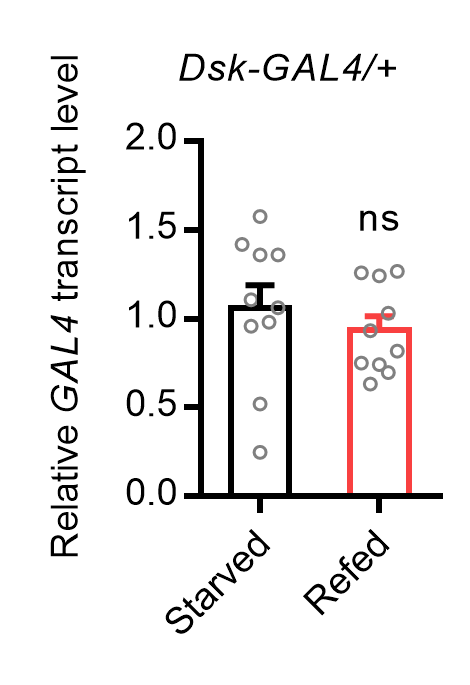

Supplement: S3 Fig — ns: not significant; Student’s t test. (TIF) [file pgen.1009724.s003.tif]

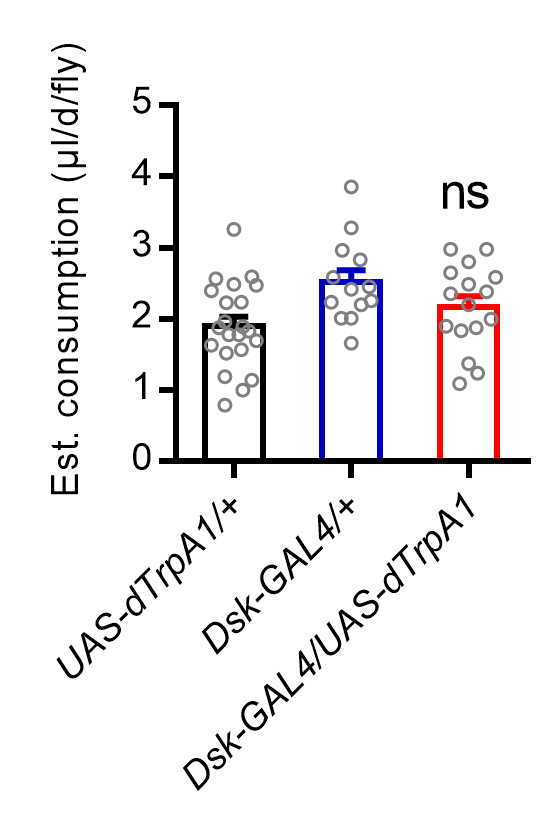

Supplement: S4 Fig — All flies were kept in CAFE tubes for 24 hours at 30°C. ns: not significant; Kruskal–Wallis test followed by Dunn’s multiple comparisons test. (TIF) [file pgen.1009724.s004.tif]

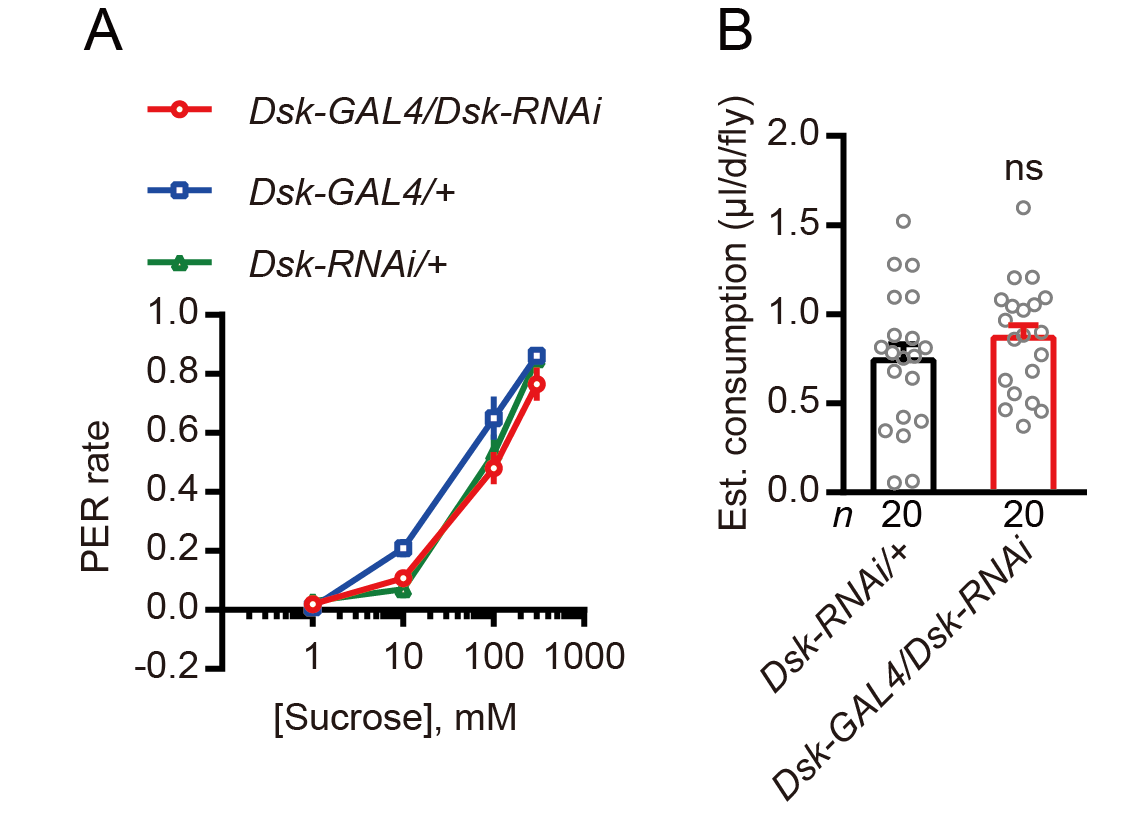

Supplement: S5 Fig — (A) Silencing the Dsk gene using the Dsk-GAL4 driver has no impact on feeding in the proboscis extension reflex (PER). n = 10 trials. ns: not significant; Mann–Whitney test. (B) Silencing the Dsk gene using the Dsk-GAL4 driver has no impact on feeding in the CAFE assay. n = 10 trials. ns: not significant; Mann–Whitney test. (TIF) [file pgen.1009724.s005.tif]

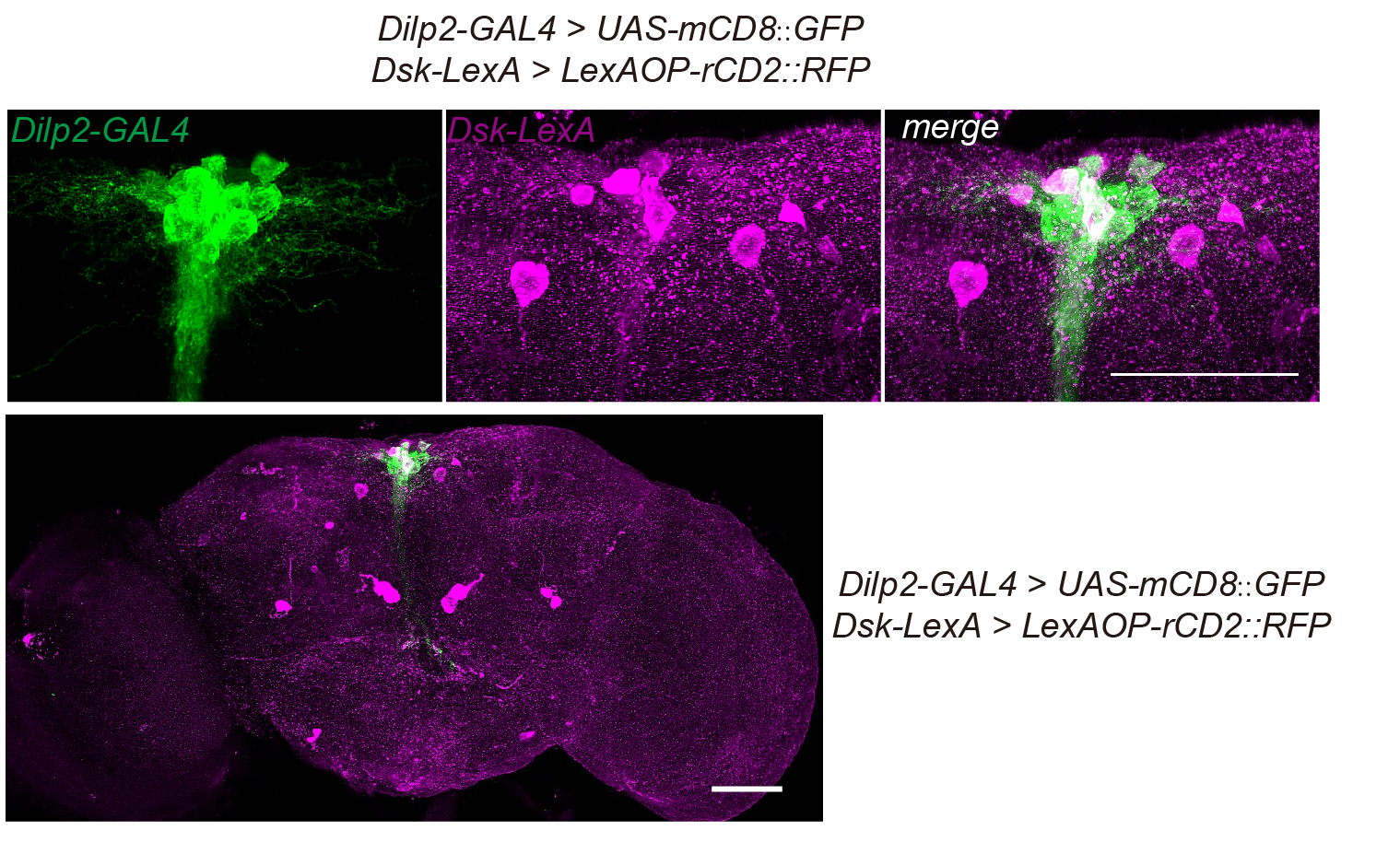

Supplement: S6 Fig — Scale bars: 50 μm. (TIF) [file pgen.1009724.s006.tif]

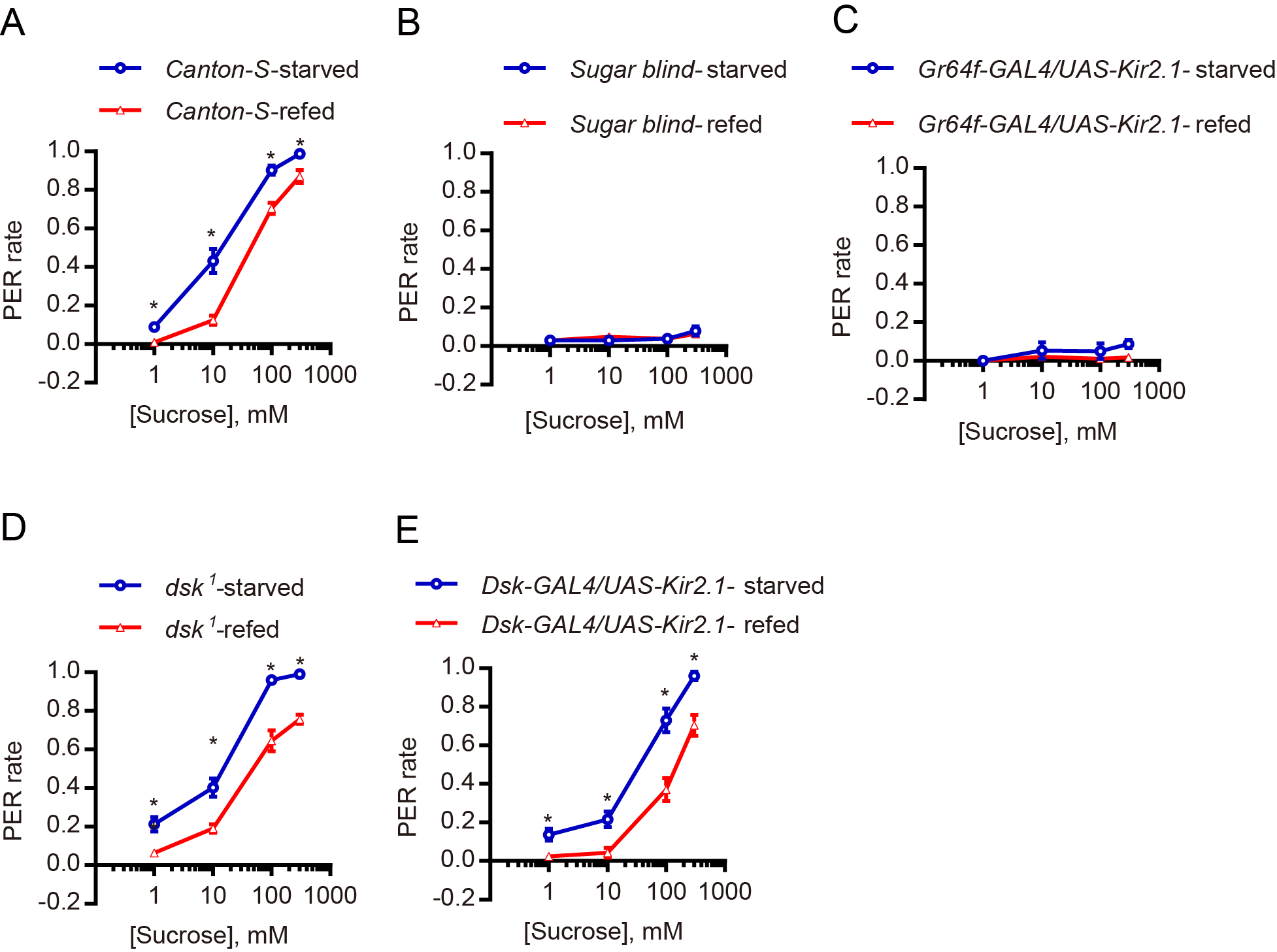

Supplement: S7 Fig — (A) Starved Canton-S showed more motivation to feed in the PER assay. n = 10 trials. *p < 0.05; Mann–Whitney test. (B and C) Sugar blind mutants and silencing sweet GRNs by expressing the Kir2.1 channel did not influence responses to starvation and re-feeding in the PER. n = 10 trials. ns, no significant; Mann–Whitney test. (D and E) Starved dsk mutants and flies with silencing of Dsk-GAL4 labeled neurons by expressing the Kir2.1 channel displayed more motivation to feed in PER. n = 10 trials. *p < 0.05; Mann–Whitney test. (TIF) [file pgen.1009724.s007.tif]

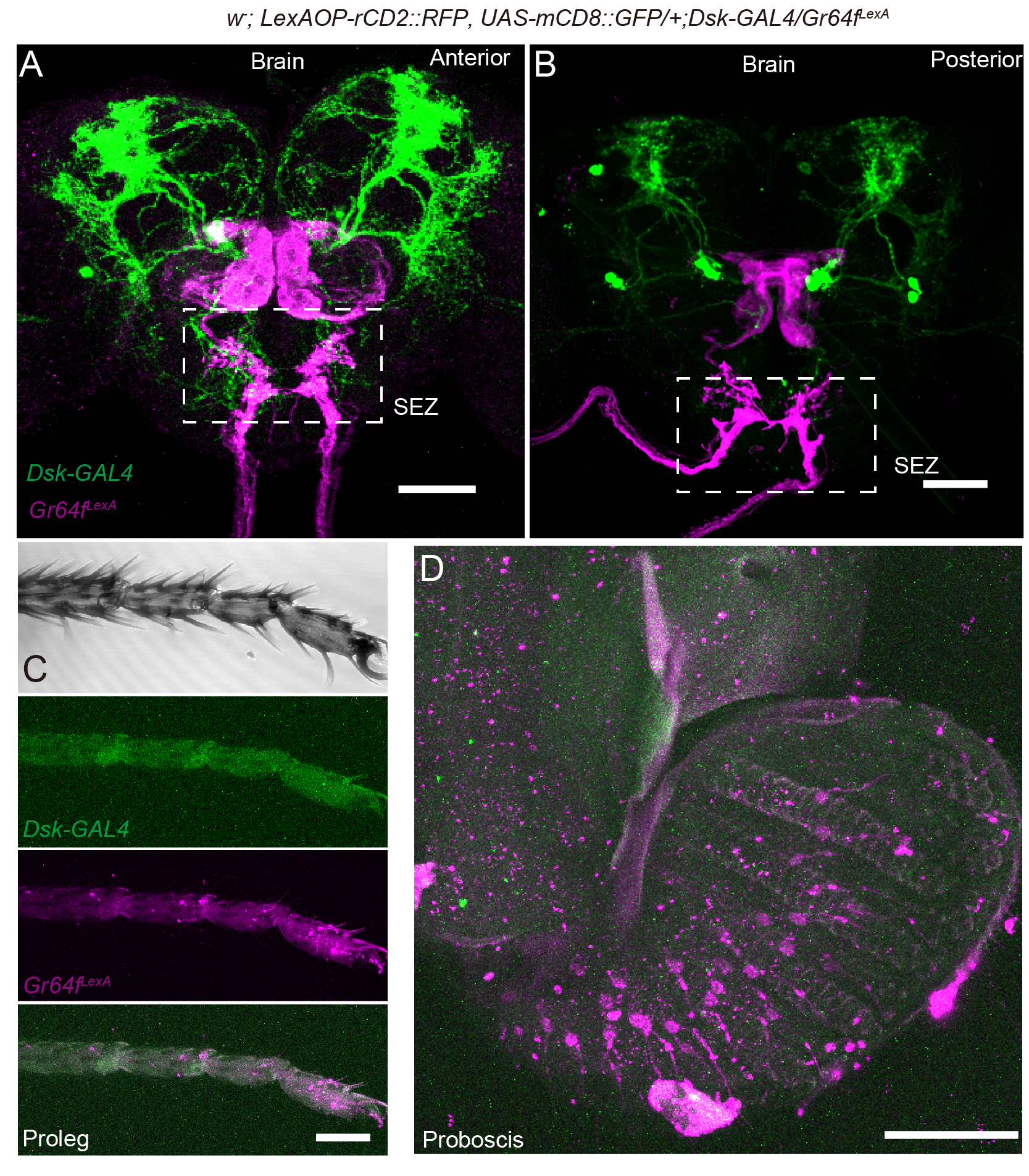

Supplement: S8 Fig — Double labeling of Dsk-GAL4-expressing neurons and Gr64fLexA-expressing sweet gustatory neurons in the Drosophila brain (A: anterior and B: posterior), proleg (C) and proboscis (D). No Dsk signal was detected on the proleg and proboscis (C and D). Scale bar: 50 μm. (TIF) [file pgen.1009724.s008.tif]

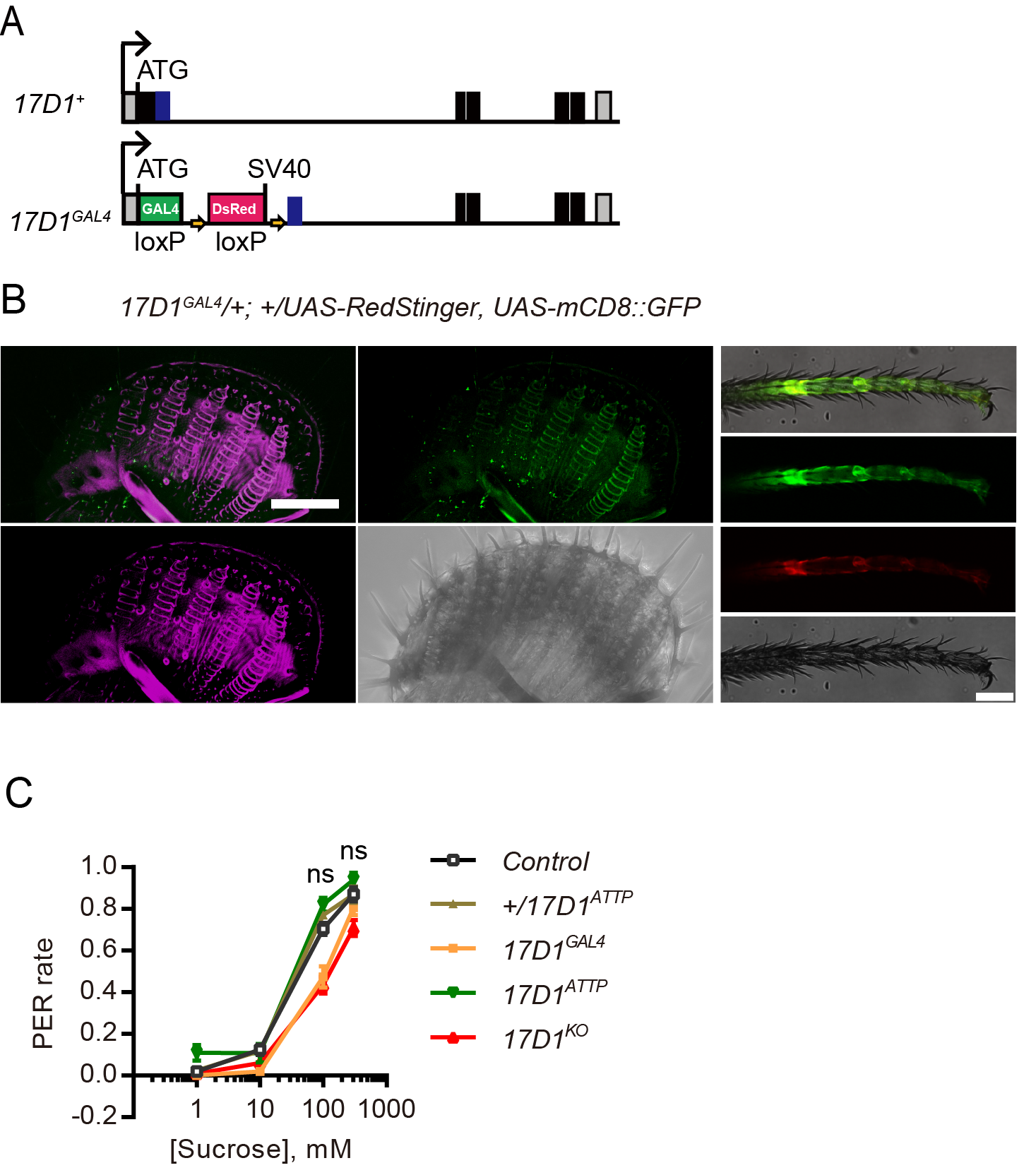

Supplement: S9 Fig — (A) Generation of knock-in of GAL4 into the CCKLR-17D1 locus. (B) No signal was detected when the 17D1GAL4 drives stinger and GFP in the proboscis and leg tarsi. Scale bar: 50 μm. (C) 17D1 mutants show no decreased motivation to feed in PER compared with control. **p < 0.01; Kruskal–Wallis test followed by Dunn’s multiple comparisons test. (TIF) [file pgen.1009724.s009.tif]

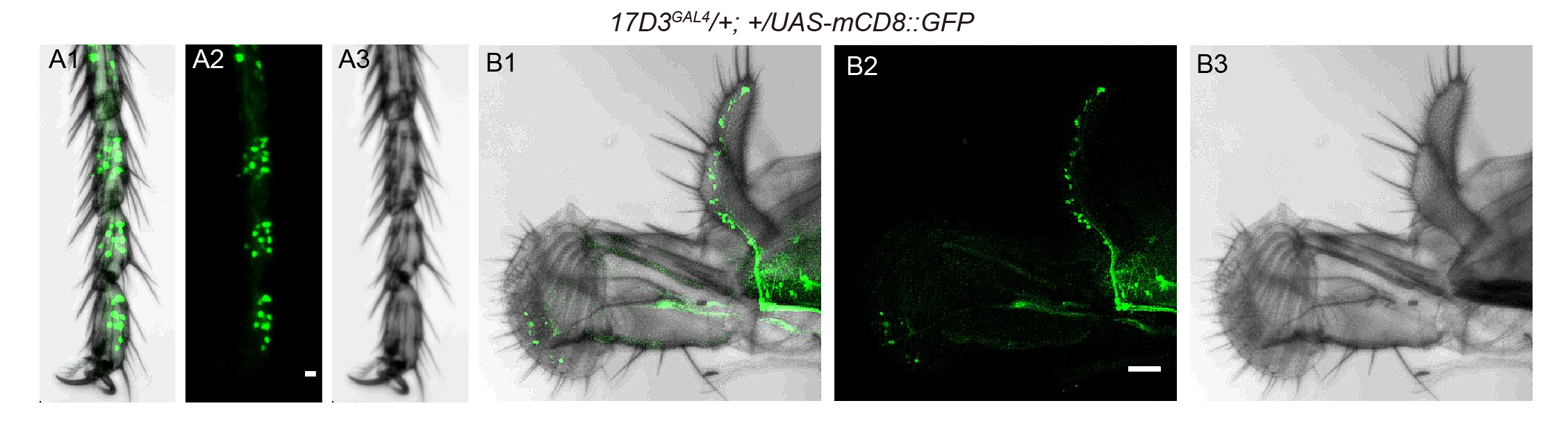

Supplement: S10 Fig — Expression pattern of 17D3GAL4 visualized by UAS-mCD8::GFP in leg tarsi (A1-A3), proboscis and maxillary palps (B1-B3). Scale bar: 50 μm. (TIF) [file pgen.1009724.s010.tif]
