# Supplementary material for: Cholecystokinin-like peptide mediates satiety by inhibiting sugar attraction
Source: PLoS Genet. 2021 Aug 16;17(8):e1009724. doi: 10.1371/journal.pgen.1009724 (PMC8366971; doi:10.1371/journal.pgen.1009724)
Supplement: S1 Table — (DOCX) [file pgen.1009724.s011.docx]

S1 Table. The primers used in this study

| Primers | Primer sequences (5’-3’) |
| --- | --- |
| For N. lugens |  |
| For real-time PCR | |
| N18SrRNA-F | CGCTACTACCGATTGAA |
| N18SrRNA-R | GGAAACCTTGTTACGACTT |
| Nlsk-F | AGCACAATGACCGCAGTTTT |
| Nlsk-R | GACGCGCCCTTATCAGTTG |
| Nlskr-F | TCGCCTCATCCCATTCCTAC |
| Nlskr-R | TGGGGAGCATAGTGACGAAG |
| Nlto-F | AAGCCCTGCAAAATCGATGC |
| Nlto-R | TGAGCCATGATCGACAGTCA |
| NlGr64f-F | TTCGGCACATCTACTGCTGG |
| NlGr64f-R | ACGCCATAGAGTGGCATGAG |
| NLGr43a-F | AATATGTTCTCTATAACCCGCA |
| NLGr43a-R | CATTTTCAAGTCTTCTCGTTCA |
| For dsRNA synthesis | |
| T7-GFP-F | **TAATACGACTCACTATAGGG**AAGGGCGAGGAGCTGTTCACCG |
| T7- GFP-R | **TAATACGACTCACTATAGGG**CAGCAGGACCATGTGATCGCGC |
| T7- Nlsk-F | **TAATACGACTCACTATAGGG**ACCGCAGTTTTGCTGACTGT |
| T7- Nlsk-R | **TAATACGACTCACTATAGGG**TTTATCGTCAGCCTCGCCG |
| T7-NlGR64f-F | **TAATACGACTCACTATAGGG**CCACCAGATGGAGGCTATGT |
| T7-NlGR64f-R | **TAATACGACTCACTATAGGG**GGTAGTAAGCCTCTGCCAGTC |
| For *Drosophila* |  |
| Primers | Primer sequences (5’-3’) |
| For real-time PCR |  |
| GAL4-F | GAAGCGAAGCCCTTTGACAC |
| GAL4-R | GAACAAACAGGCCAGTGAGC |
| DmRp49-F | CACACCAAATCTTACAAAATGTGTGA |
| DmRp49-R | AATCCGGCCTTGCACATG |
| DmTo-F | AGTCCCGTGGGCATAACTCT |
| DmTo-R | GATTGCCCTGACGTTAACCAT |
| DmGr64f-F | TCACGAGAGTGCCCGAAAAA |
| DmGr64f-R | CCGGCATCATGGCAAAGAAC |
| CCKLR17D1-F | GCTACGTGAGCGACAATGAA |
| CCKLR17D1-R | AGCCCACGTACTCGTACACC |
| CCKLR17D3-F | ACGCGTACCCTGTACGTAGG |
| CCKLR17D3-R | GGTCTCGTTGTCAAGGTGGT |
| DSK-F | CCGATCCCAGCGCAGACGAC |
| DSK-R | TGGCACTCTGCGACCGAAGC |
