# Supplementary material for: Cholecystokinin-like peptide mediates satiety by inhibiting sugar attraction
Source: PLoS Genet. 2021 Aug 16;17(8):e1009724. doi: 10.1371/journal.pgen.1009724 (PMC8366971; doi:10.1371/journal.pgen.1009724)
Supplement: S2 Table — (DOCX) [file pgen.1009724.s012.docx]

S2 Table Summary of sequence assembly after RNA-seq of silence *Nlsk* gene

| Sample | Library | Raw reads | Clean reads | Clean bases | Error rate（%） | Q20（%） | Q30(%) | GC content (%) |
| --- | --- | --- | --- | --- | --- | --- | --- | --- |
| dsgfp_1 | RRAS51407-V | 44336972 | 43249838 | 6.49G | 0.03 | 96.89 | 92.33 | 50.99 |
| dsgfp_2 | RRAS51408-V | 48527522 | 47263488 | 7.09G | 0.03 | 96.99 | 92.46 | 49.68 |
| dsgfp_3 | RRAS51409-V | 47718054 | 46533320 | 6.98G | 0.03 | 96.98 | 92.3 | 49.12 |
| dsgfp_4 | RRAS51410-V | 53082812 | 51971334 | 7.8G | 0.03 | 97.37 | 93.23 | 49.42 |
| dsNlsk_1 | RRAS51411-V | 55804370 | 54540464 | 8.18G | 0.03 | 97.2 | 92.92 | 49.76 |
| dsNlsk_2 | RRAS51412-V | 49524442 | 46988924 | 7.05G | 0.03 | 97.14 | 92.77 | 51.37 |
| dsNlsk_3 | RRAS51413-V | 51935656 | 49617706 | 7.44G | 0.03 | 97.17 | 92.91 | 51.53 |
| dsNlsk_4 | RRAS51414-V | 46468110 | 45076184 | 6.76G | 0.03 | 97.21 | 92.89 | 50.97 |
