# Supplementary material for: Cholecystokinin-like peptide mediates satiety by inhibiting sugar attraction
Source: PLoS Genet. 2021 Aug 16;17(8):e1009724. doi: 10.1371/journal.pgen.1009724 (PMC8366971; doi:10.1371/journal.pgen.1009724)
Supplement: S3 Table — (DOCX) [file pgen.1009724.s013.docx]

S3 Table Number of reads sequenced and mapped to the genome of silence *Nlsk* gene.

| Sample | Total reads | Total map | Unique map | Multi map | Read1 map | Read2 map | Positive map | Negative map | Splice map | Unsplice map | Proper map |
| --- | --- | --- | --- | --- | --- | --- | --- | --- | --- | --- | --- |
| dsgfp_1 | 43249838 | 32865301  (75.99%) | 27998713  (64.74%) | 4866588  (11.25%) | 14057164  (32.5%) | 13941549  (32.23%) | 14051500  (32.49%) | 1394721  3(32.25%) | 13718129  (31.72%) | 14280584  (33.02%) | 24480394  (56.6%) |
| dsgfp_2 | 47263488 | 36033664  (76.24%) | 29286851  (61.97%) | 6746813  (14.27%) | 14672448  (31.04%) | 14614403  (30.92%) | 14695050  (31.09%) | 14591801  (30.87%) | 13830025  (29.26%) | 15456826  (32.7%) | 25640042  (54.25%) |
| dsgfp_3 | 46533320 | 36246440  (77.89%) | 30807836  (66.21%) | 5438604  (11.69%) | 15465977  (33.24%) | 15341859  (32.97%) | 15468587  (33.24%) | 15339249  (32.96%) | 14750407  (31.7%) | 16057429  (34.51%) | 27142550  (58.33%) |
| dsgfp_4 | 51971334 | 40291559  (77.53%) | 33210180  (63.9%) | 7081379  (13.63%) | 16661424  (32.06%) | 16548756  (31.84%) | 16666326  (32.07%) | 16543854  (31.83%) | 16129527  (31.04%) | 17080653  (32.87%) | 29404944  (56.58%) |
| dsNlsk_1 | 54540464 | 40918433  (75.02%) | 33277750  (61.01%) | 7640683  (14.01%) | 16651239  (30.53%) | 16626511  (30.48%) | 16698179  (30.62%) | 16579571  (30.4%) | 15415805  (28.26%) | 17861945  (32.75%) | 29124832  (53.4%) |
| dsNlsk_2 | 46988924 | 35651723  (75.87%) | 30198817  (64.27%) | 5452906  (11.6%) | 15127179  (32.19%) | 15071638  (32.07%) | 15177396  (32.3%) | 15021421  (31.97%) | 15085855  (32.11%) | 15112962  (32.16%) | 26767568  (56.97%) |
| dsNlsk_3 | 49617706 | 37393064  (75.36%) | 31706204  (63.9%) | 5686860  (11.46%) | 15862900  (31.97%) | 15843304  (31.93%) | 15913901  (32.07%) | 15792303  (31.83%) | 15286908  (30.81%) | 16419296  (33.09%) | 28154294  (56.74%) |
| dsNlsk_4 | 45076184 | 34968110  (77.58%) | 29598797  (65.66%) | 5369313  (11.91%) | 14837064  (32.92%) | 14761733  (32.75%) | 14866665  (32.98%) | 14732132  (32.68%) | 15139320  (33.59%) | 14459477  (32.08%) | 26499278  (58.79%) |
